# Matrix-assisted laser desorption/ionization time-of-flight mass spectrometry for differential identification of adult *Schistosoma* worms

**DOI:** 10.1186/s13071-022-05604-0

**Published:** 2023-01-19

**Authors:** Jurena Christiane Ebersbach, Marcello Otake Sato, Matheus Pereira de Araújo, Megumi Sato, Sören L. Becker, Issa Sy

**Affiliations:** 1 Institute of Medical Microbiology and Hygiene, Saarland University, Homburg, Germany; 2 Laboratory of Tropical Medicine and Parasitology, Dokkyo Medical University, Mibu, Tochigi Japan; 3 Graduate School of Health Sciences, Niigata University, Niigata, Japan; 4 Swiss Tropical and Public Health Institute, Allschwil, Switzerland; 5 University of Basel, Basel, Switzerland

**Keywords:** Identification, *Schistosoma mansoni*, *Schistosoma japonicum*, Helminth, Matrix-assisted laser desorption/ionization-time of flight mass spectrometry, Trematode, Storage media, Machine learning

## Abstract

**Background:**

Schistosomiasis is a major neglected tropical disease that affects up to 250 million individuals worldwide. The diagnosis of human schistosomiasis is mainly based on the microscopic detection of the parasite’s eggs in the feces (i.e., for *Schistosoma mansoni* or *Schistosoma japonicum*) or urine (i.e., for *Schistosoma haematobium*) samples. However, these techniques have limited sensitivity, and microscopic expertise is waning outside endemic areas. Matrix-assisted laser desorption/ionization time-of-flight (MALDI-TOF) mass spectrometry (MS) has become the gold standard diagnostic method for the identification of bacteria and fungi in many microbiological laboratories. Preliminary studies have recently shown promising results for parasite identification using this method. The aims of this study were to develop and validate a species-specific database for adult *Schistosoma* identification, and to evaluate the effects of different storage solutions (ethanol and RNA*later*) on spectra profiles.

**Methods:**

Adult worms (males and females) of *S. mansoni* and *S. japonicum* were obtained from experimentally infected mice. Species identification was carried out morphologically and by cytochrome oxidase 1 gene sequencing. Reference protein spectra for the creation of an in-house MALDI-TOF MS database were generated, and the database evaluated using new samples. We employed unsupervised (principal component analysis) and supervised (support vector machine,* k*-nearest neighbor, Random Forest, and partial least squares discriminant analysis) machine learning algorithms for the identification and differentiation of the *Schistosoma* species.

**Results:**

All the spectra were correctly identified by internal validation. For external validation, 58 new *Schistosoma* samples were analyzed, of which 100% (58/58) were correctly identified to genus level (log score values ≥ 1.7) and 81% (47/58) were reliably identified to species level (log score values ≥ 2). The spectra profiles showed some differences depending on the storage solution used. All the machine learning algorithms classified the samples correctly.

**Conclusions:**

MALDI-TOF MS can reliably distinguish adult *S. mansoni* from *S. japonicum*.

**Graphical Abstract:**

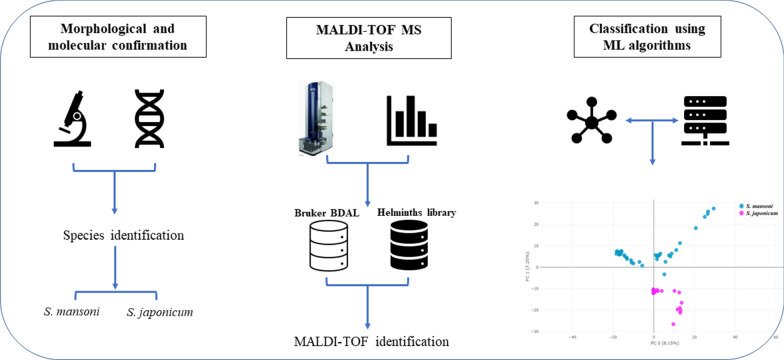

**Supplementary Information:**

The online version contains supplementary material available at 10.1186/s13071-022-05604-0.

## Background

In tropical and subtropical areas, schistosomiasis is a major cause of morbidity; it is one of the clinically most relevant water-borne parasitic diseases worldwide [1, 2], and an estimated 250 million people are infected with it in a total of 78 countries [3, 4]. Schistosomiasis can cause severe allergy-like reactions in initial disease stages (e.g., Katayama syndrome), while it can later lead to significant long-term morbidity, e.g., diarrhea, hematuria, depending on the site of infection, and considerable complications (e.g., hepatic fibrosis, bladder cancer) [5]. *Schistosoma* belong to the family Schistosomatidae, with three main species infecting humans: *Schistosoma mansoni*, occurring mainly in the sub-Saharan region and South America; *Schistosoma japonicum*, mainly found in China, the Philippines, and Sulawesi (Indonesia); and *Schistosoma haematobium*, found in Africa and parts of the Middle East. Active infections are most frequently found in schoolchildren and young adults [4].

*Schistosoma* has a complex life cycle that requires an intermediate host (freshwater snail). When they come into contact with water, *Schistosoma* eggs hatch and release miracidia. The miracidia penetrate snail tissues and develop into cercariae, which are released into the water and constitute the infective stage. Humans and other mammals (definitive hosts) can become infected through contact with contaminated freshwater. The schistosomulae successively enter the lungs, the heart, and the liver via the venous circulation, and leave the liver via the portal system once the maturation process is complete. Male and female adult worms live in the mesenteric veins, where they copulate, and lay their eggs in the small venules of the portal and perivesical systems [3]. The transmission cycle continues when hosts infected with schistosomiasis contaminate freshwater sources with their excreta, which contain the parasite’s eggs [6, 7].

Morphological identification of eggs from stool or urine samples using microscopy is the most widely employed standard technique for the diagnosis of schistosomiasis [3]. However, this method is laborious and has limited sensitivity, especially for infections of light intensity [8]. Hence, several other tests have been developed, e.g., using polymerase chain reaction (PCR) [9], and serology [8]. However, these have shortcomings with regard to their field applicability, diagnostic accuracy and/or accessibility—for example, no commercially available PCR test kits are available for *Schistosoma* diagnostics [10]. The serological method is very sensitive, but its specificity is poor, as it cannot distinguish active from past infections [2, 7].

Matrix-assisted laser desorption/ionization time-of-flight (MALDI-TOF) mass spectrometry (MS) is the standard diagnostic method for the identification of culture-grown bacteria, mycobacteria, and fungi in clinical laboratories. Recently, this technique has also been investigated in clinical research as a new tool for the identification of mosquitoes [11, 12], ticks [13] and other arthropods [14] and, to a lesser extent, intestinal parasites (e.g., helminths) [8]. Species identification through MALDI-TOF MS is based on the comparison of protein spectra of unknown, independent samples to reference spectra of well-characterized species that are held in a MALDI database. Compared to the other identification techniques, the advantages of this method are its reliability and rapidity, cost-effectiveness due to the reagents used, its availability in many microbiological laboratories, particularly in high-income countries, where expertise in the identification and differentiation of parasitic elements is waning, and its ease of use once a database (which includes the reference spectra of the targeted microorganisms) is available. However, there are scant data on trematode identification from MALDI-TOF MS. Against this background, the aims of this study were (i) to employ MALDI-TOF MS for the identification of adult *Schistosoma* species, even though adult worms are not used in human diagnosis, as this allowed us to evaluate the potential of this technology for future applications in clinical diagnostics using available materials (i.e., egg samples from stool or urine); (ii) to assess the accuracy of identification for the differentiation of adult *S. mansoni* from *S. japonicum* (interspecies classification); and (iii) to evaluate the effect of different storage media [RNA*later*, and 70% (*v*/*v*) ethanol] on samples by using MALDI-TOF MS coupled with machine learning (ML) classification algorithms.

## Methods

### Ethics statement

All the experimental procedures involving animals were conducted in strict accordance with the Institutional Animal Care Guidelines and approved by the Ethical Committee for Animal Experimentation of Dokkyo Medical University under number 1307.

### Origin of the parasitic material

Adult worms of *S. mansoni* (Puerto Rican strain) and *S. japonicum* (Japanese Yamanashi strain) used in this study were obtained from experimentally infected BALBc mice. The infected animals were maintained at the animal facility of the Laboratory of Tropical Medicine and Parasitology of Dokkyo Medical University. For the current investigation, a total of 62 adults of *Schistosoma* spp*.* were obtained from either the mesenteric or portal veins of the experimental animals and washed with PBS several times, before being morphologically identified and stored in two different media: 70% (*v*/*v*) ethanol; RNA*late*r (Invitrogen, USA), which is an aqueous, nontoxic, tissue and cell collection reagent that stabilizes and protects RNA and proteins in intact, unfrozen tissue and cell samples. Sample identification was further confirmed by DNA sequencing (see “Molecular identification of *Schistosoma* spp. samples” section). Forty samples were identified as *S. mansoni*, while the remaining 22 corresponded to *S. japonicum*. Of the 40 *S. mansoni* isolates, 19 (seven mixed males/females, six males, six females) were placed in 70% (*v*/*v*) ethanol, and 21 (11 mixed males/females, five males, five females) in RNA*later*. For the 22 *S. japonicum* isolates, 11 (five mixed males/females, three males, three females) were stored in 70% (*v*/*v*) ethanol and 11 (five mixed males/females, three males, three females) in RNA*later*. All the samples were stored at −40 °C before being transferred to the Institute of Medical Microbiology and Hygiene (Homburg, Germany), where they were stored at −20 °C pending further examination.

### Molecular identification of *Schistosoma* spp. samples

For molecular confirmation of the individual *Schistosoma* species obtained from the mice, genomic DNA of two adult worms from each experimental infection was extracted using a commercially available DNA extraction kit (DNeasy Blood & Tissue Kit; QIAGEN, USA) according to the manufacturer’s instructions. Genomic DNA from morphologically identified *S. mansoni* or *S. japonicum* was used for the amplification of cytochrome oxidase 1 (*COX1*) by PCR using primer pairs specific for *S. mansoni* (TCCTTTATCAATTTGAGAGG/CR: CCAACCATAAACATATGATG) and *S. japonicum* (CCGTTTTTTTTGAGTATGAG/CR: CCAACCATAAACATATGATG), with an expected length of 479 and 614 base pairs, respectively [15]. The reactions were carried out in a final volume of 50 μL, using KOD One PCR Master Mix (Toyobo, Japan) with 10 μmol each of the forward and reverse primers and 1.0 μL (approximately 10 ng/μL) genomic DNA. Cycling conditions for the PCR consisted of a 2-min denaturation step at 94 °C, followed by 35 cycles of denaturation at 98 °C for 10 s, annealing at 58 °C for 30 s, and extension at 68 °C for 30 s, and final extension at 72 °C for 7 min. PCR products were detected in 2% agarose gel stained with 1% ethidium bromide using Tris–borate–ethylenediaminetetraacetic acid buffer. The PCR products were purified using a commercial DNA purification kit (QIAquick Gel Extraction Kit; QIAGEN, Hilden, Germany) following the manufacturer’s protocol. Purified PCR products were sequenced in a 3130xl Genetic Analyzer (Applied Biosystems, USA). Sequences were assembled using Molecular Evolutionary Genetics Analysis version 10 [16] and a Nucleotide Basic Local Alignment Search Tool (BLASTn) search (https://blast.ncbi.nlm.nih.gov) was performed for the confirmation of sequence identity of the generated consensus sequences. A phylogenetic tree based on the analysis of *COX1* gene sequences was constructed after 1000 bootstrap replications, using the maximum likelihood method and the Hasegawa-Kishono-Yano model. The outgroup sequence *S. haematobium* (accession ID: ON237718), as well as other *COX1* sequences of *S. mansoni* (accession IDs: MK171834, MF919418, and MG562513) and *S. japonicum* (accession IDs: KU196387, KU196397, and KU196417) were retrieved from GenBank and added to the analysis.

### MALDI-TOF MS analysis

#### Sample preparation

Adult worm samples were removed from the storage solution and dried at room temperature in a biosafety cabinet for about 5 min, to allow for the evaporation of organic solvents prior to the subsequent analyses.

#### Protein extraction

A previously employed protocol was adapted and applied for protein extraction of the adult *Schistosoma* samples [10]. In brief, adult worms were manually crushed in 300 µL liquid chromatography-mass spectrometry (LC–MS) grade water (Merck, Darmstadt, Germany). Then, 900 µL of 100% (*v*/*v*) absolute ethanol (Merck, Darmstadt, Germany) was added before mixing by vortexing. The mixture was centrifuged at 18,312 × *g* for 2 min, and the supernatant was discarded. After having completely dried the pellet, it was resuspended in 20 µL of 70% (*v*/*v*) formic acid and mixed by vortexing. Finally, 20 µL acetonitrile was added before mixing again.

#### MALDI target plate preparation and measurements

The protein extracts (see the “Protein extraction” section) mixed with formic acid and acetonitrile were centrifuged at 18,312 × *g* for 2 min. One microliter of the clear supernatant was spotted onto the MALDI-TOF MS target plate (Bruker Daltonics, Bremen, Germany) then allowed to dry completely before covering it with 1 µL of α-cyano-4-hydroxycinnamic acid matrix solution (Bruker Daltonics) composed of saturated α-cyano-4-hydroxycinnamic acid, 50% (*v*/*v*) acetonitrile, 2.5% (*v*/*v*) trifluoroacetic acid and 47.5% (*v*/*v*) LC–MS grade water. The protein extracts of each sample were spotted onto the MALDI-TOF MS target plate at eight different spots, and each spot was measured four times to assure reproducibility. Hence, a total of 32 raw spectra per sample were generated using FlexControl^®^ software version 3.4 (Bruker Daltonics). The bacterial test standard (Bruker Daltonics), which is an extract of *Escherichia coli* spiked with two high molecular weight proteins, was used to calibrate the mass spectrometer. After drying at room temperature, the MALDI target plate was placed into a Microflex LT Mass Spectrometer (Bruker Daltonics) for the measurements.

#### MALDI-TOF MS parameters

Measurements were performed using the AutoXecute algorithm implemented in FlexControl software version 3.4. For each spot, a total of 240 laser shots (40 shots each, six random positions) were carried out automatically to generate protein mass profiles in linear positive ion mode with a laser frequency of 60 Hz, a voltage of 20 kV, and a pulsed ion extraction of 180 ns. Mass charge ratios range (m/z) were measured between 2 and 20 kDa.

#### Spectra inspection and creation of reference spectra

Raw spectra were visualized using FlexAnalysis software version 3.4 (Bruker Daltonics). The spectra were edited, i.e., all flatlines and outlier peaks were removed, intensities were smoothed, and peak shifts within replicated spectra were set at 300 p.p.m. After this editing step, spectra of four mixed (males/females) adult worm samples (two *S. mansoni*, two *S. japonicum*) from both of the storage media (RNA*later*, ethanol), comprising at least 27 remaining spectra each, were randomly selected for the creation of reference spectra (main spectra profiles; MSPs). These MSPs were created using the automatic function of MALDI Biotyper Compass Explorer^®^ software version 3 (Bruker Daltonics). The newly created MSPs of both *Schistosoma* species were included in a previously developed in-house MALDI-TOF MS database for helminth identification, which already contained MSPs from different helminths such as cestodes (e.g., *Taenia saginata*) and trematodes (e.g., *Fasciola* spp.) [10, 17].

#### Database validation

To verify the purity and check if any of the spectra matched bacterial spectra, all the acquired spectra were tested against the commercially available, official BDAL database released by Bruker Daltonics for the identification of bacteria and fungi. The newly expanded in-house helminth database was subjected to two different validation procedures. First, to an internal validation procedure, in which all raw spectra of *Schistosoma* spp. obtained during the MSP process were tested to verify whether it was possible to identify them from existing spectra in the database. Second, to an external validation procedure, where spectra from the 58 remaining, independent, adult *Schistosoma* specimens were investigated to assess whether they could be reliably identified from the database. For this purpose, spectra were examined using a combination of the official BDAL database (Bruker Daltonics) and our in-house helminth database. The reliability of the identification was interpreted by log score values (LSVs) generated for each identification result. We used the scoring system recommended by the manufacturer for bacteria identification (i.e., an LSVs of 1.70 was considered the threshold for reliable identification; LSVs between 1.70 and 1.99 indicated reliable identification at the genus level, and LSVs equal to or higher than 2.0 were interpreted as indicating reliable species identification) [10].

### Classification and comparisons analysis

#### Pre-processing parameters

A total of 1657 edited spectra were exported into the free online software Clover MS Data Analysis (https://platform.clovermsdataanalysis.com/, Clover BioSoft, Granada, Spain) (last accessed May 2022) for further investigation. Default parameters were used during pre-processing [18]. A Savitzky–Golay filter (window length, 11; order 3 polynomial) was applied to smooth the spectra, and the baseline was removed using the top-hat filter method (factor 0.02). To obtain one average spectrum per sample for use in the classification and comparisons analysis, replicated spectra were aligned using the following parameters: allowed shift, medium; constant tolerance, 0.2 Da; linear tolerance, 2000 p.p.m.

#### Classification using ML algorithms

Peak matching was performed to generate a peak matrix from pre-processed spectra that were used for comparison analysis. Total ion current normalization was applied, followed by a threshold method (factor 0.01), where peaks with an intensity below 1% of the maximum intensity were not considered; the constant tolerance was 0.5 Da and the linear tolerance 500 p.p.m. [18].

Classification analysis was carried out at two levels. First, at the interspecies level, where all isolates were investigated to distinguish *S. mansoni* from *S. japonicum.* Second, at the intraspecies level, where samples of the same species were compared to assess the discrimination related to the effect of the storage solutions [70% (*v*/*v*) ethanol, and RNA*later*]. Unsupervised [principal component analysis (PCA), hierarchical clustering], and supervised ML algorithms were used to assess the classification. A PCA is a dimensionality reduction algorithm (it reduces a high-dimensional dataset to a set of coordinates to allow for better visualization of different clusters and relationships among specimens for the identification of subgroups) and provides information about the “true” nature of a dataset [19]. The hierarchical clustering was performed using the Chebyshev method for distance calculation and the complete method for the metric. For the supervised ML methods, four widely used algorithms for MALDI-TOF mass spectra analysis [linear support vector machine (SVM), partial least squares-discriminant analysis (PLS-DA), Random Forest (RF), and* k*-nearest neighbors (KNN)] were evaluated [20]. The* k*-fold cross-validation method (*k* = 10) was used for the internal validation. A confusion matrix (generating values such as accuracy, specificity, sensitivity, F1 score, positive prediction value or precision, and negative prediction value), as well as the area under the receiver operating characteristic curve, and the area under the precision recall curve, were used as performance metrics of the supervised ML algorithms.

## Results

### Molecular analysis

**Fig. 1:**
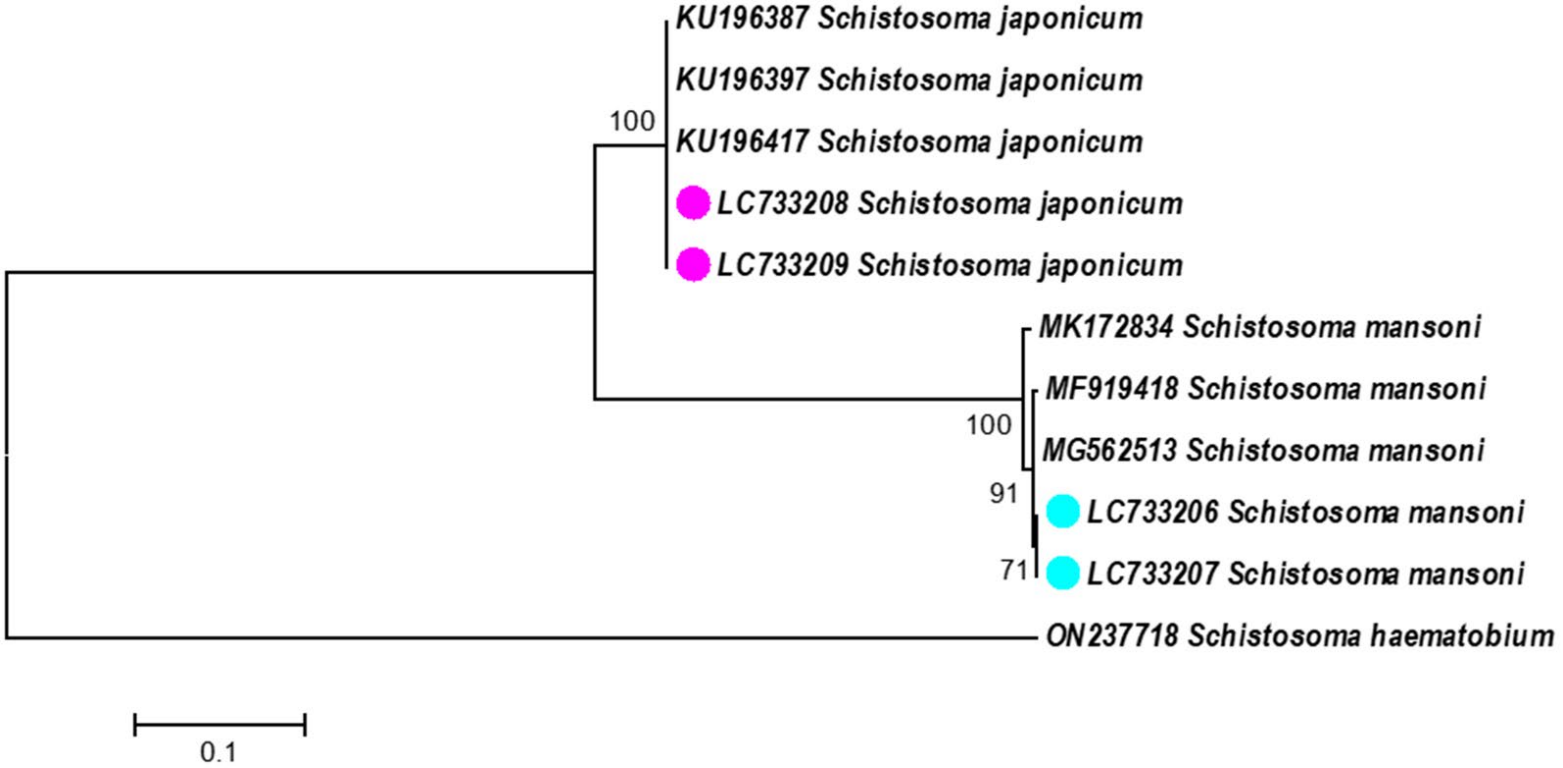
Phylogenetic tree based on cytochrome oxidase 1 gene (*COX1*) sequences by the maximum likelihood method. The values on the nodes represent bootstrap values. The colored circles (light blue for *Schistosoma mansoni*, violet for *Schistosoma japonicum*) indicate isolates analyzed in this study

The morphological identification of *S. mansoni* and *S. japonicum* was confirmed from the sequences obtained from the four isolates. Partial sequences of the *COX1* gene were deposited in the GenBank database with the consecutive accession IDs LC733206–LC733209. The phylogenetic tree based on the *COX1* sequences revealed two main groups corresponding to the two species. Bootstrap values obtained for both clades (100%) indicated high similarities with the sequences recovered from GenBank (Fig. 1).

### Spectra analysis and purity control

**Fig. 2:**
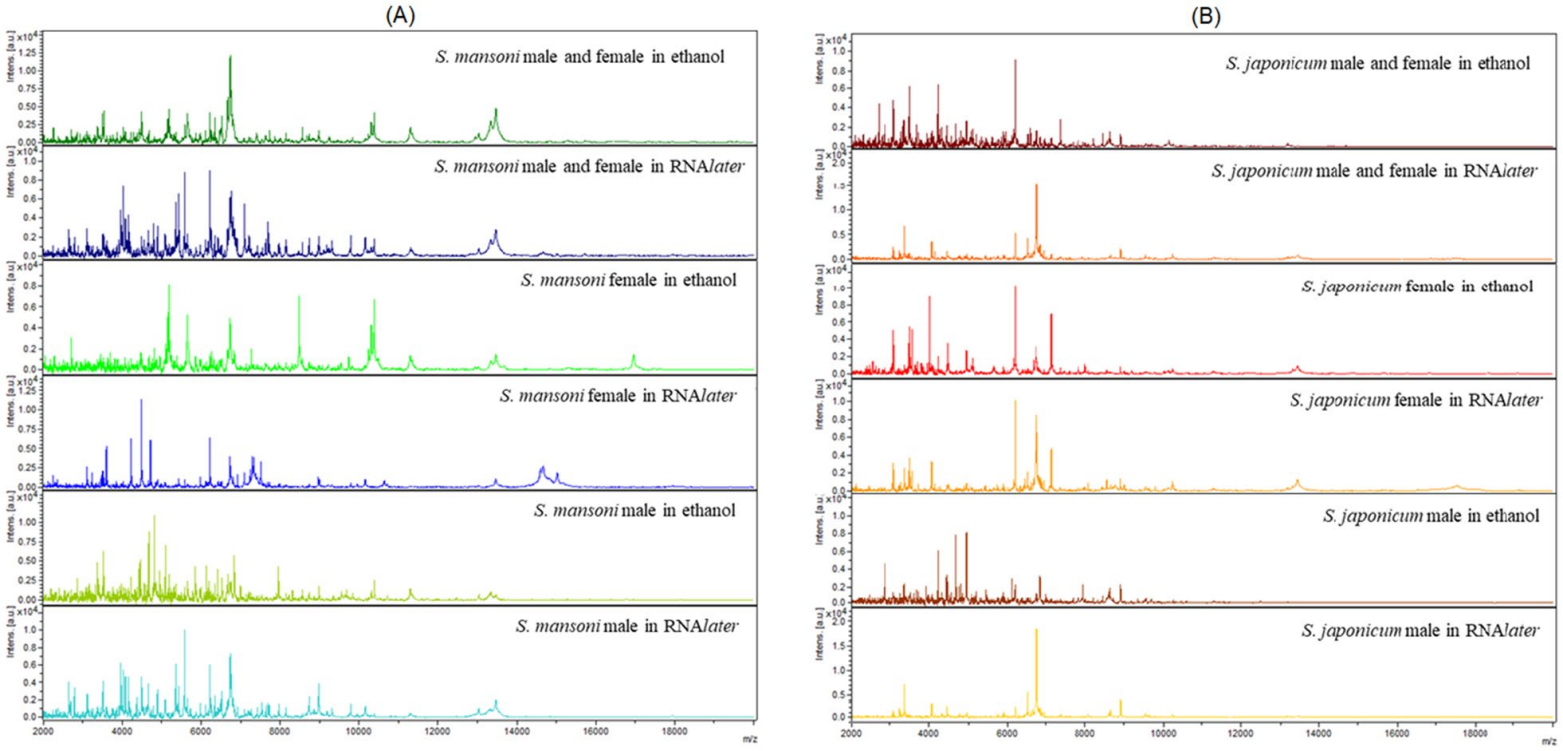
Representative matrix-assisted laser desorption/ionization time-of-flight (MALDI-TOF) mass spectrometry (MS) spectra profiles of adult **a** *Schistosoma mansoni* and **b** *Schistosoma japonicum* stored in 70% (*v*/*v*) ethanol and RNA*later. x*-axis Mass-to-charge (m/z) ratio;* y*-axis intensity values in arbitrary units

Spectra with high intensities were generated for *Schistosoma* samples stored in ethanol, as well for those stored in RNA*later*. The spectra profiles obtained by MALDI-TOF MS of different samples of *S. mansoni* and *S. japonicum* were unique for each of the two species. However, a few differences related to the preservation media were also observed (Fig. 2). None of the spectra matched those of bacterial species included in the database, which indicated that none of the samples were contaminated with any of these bacteria (Additional file 1: Table S1).

### In-house database validation

All the spectra were identified correctly through the internal validation of our database, i.e., 100% correct identification (Table 1). For the external validation, 58 “new” *Schistosoma* samples were analyzed; correct identification at the genus level (LSVs ≥ 1.7) was achieved for all samples (100%), while 81% (47/58) were correctly identified at the species level (LSVs ≥ 2.0). Of note, more *S. japonicum* (85%) than *S. mansoni* (79%) samples were correctly identified at the species level (Table 1). There was no species mismatch for any of the samples, even within an LSV range of 1.7–2.0.

**Table 1:** Internal and external validation of the identification of *Schistosoma* specimens stored in different media [70% (*v*/*v*) ethanol, RNA*later*], using a newly expanded in-house database, implemented in MBT Compass Explorer software (Bruker Daltonics, Bremen, Germany)

Internal validation					
Species	Preservation media	Number of samples	Identification results		
			Number of spectra	LSV ≥ 1.7	LSV ≥ 2.0
				Mixed adults (males/females)	Mixed adults (males/females)
*Schistosoma mansoni*	Ethanol	1	31	31/31 (100%)	31/31 (100%)
	RNA*later*	1	27	27/27 (100%)	27/27 (100%)
*Schistosoma japonicum*	Ethanol	1	29	29/29 (100%)	29/29 (100%)
	RNA*later*	1	32	32/32 (100%)	32/32 (100%)
	Total	4	119	119/119 (100%)	119/119 (100%)

External validation										
Species	Preservation media	Number of samples	Identification results							
			LSV ≥ 1.7				LSV ≥ 2.0			
			Mixed adults (males/females)	Females	Males	% Correct	Mixed adults (males/females)	Females	Males	% Correct
*S. mansoni*	Ethanol	18	6/6 (100%)	6/6 (100%)	6/6 (100%)	18/18 (100%)	6/6 (100%)	5/6 (83.3%)	6/6 (100%)	11/18 (61.1%)
	RNA*later*	20	10/10 (100%)	5/5 (100%)	5/5 (100%)	20/20 (100%)	10/10 (100%)	4/5 (80%)	5/5 (100%)	19/20 (95%)
*S. japonicum*	Ethanol	10	4/4 (100%)	3/3 (100%)	3/3 (100%)	10/10 (100%)	4/4 (100%)	0/3 (0%)	3/3 (100%)	7/10 (70%)
	RNA*later*	10	4/4 (100%)	3/3 (100%)	3/3 (100%)	10/10 (100%)	4/4 (100%)	3/3 (100%)	3/3 (100%)	10/10 (100%)
Total		58	26/26 (100%)	17/17 (100%)	17/17 (100%)	58/58 (100%)	24/24 (100%)	12/17 (70.6%)	11/17 (64.7%)	47/58 (81%)

*LSV* Log score value

### Interspecies classification

**Fig. 3:**
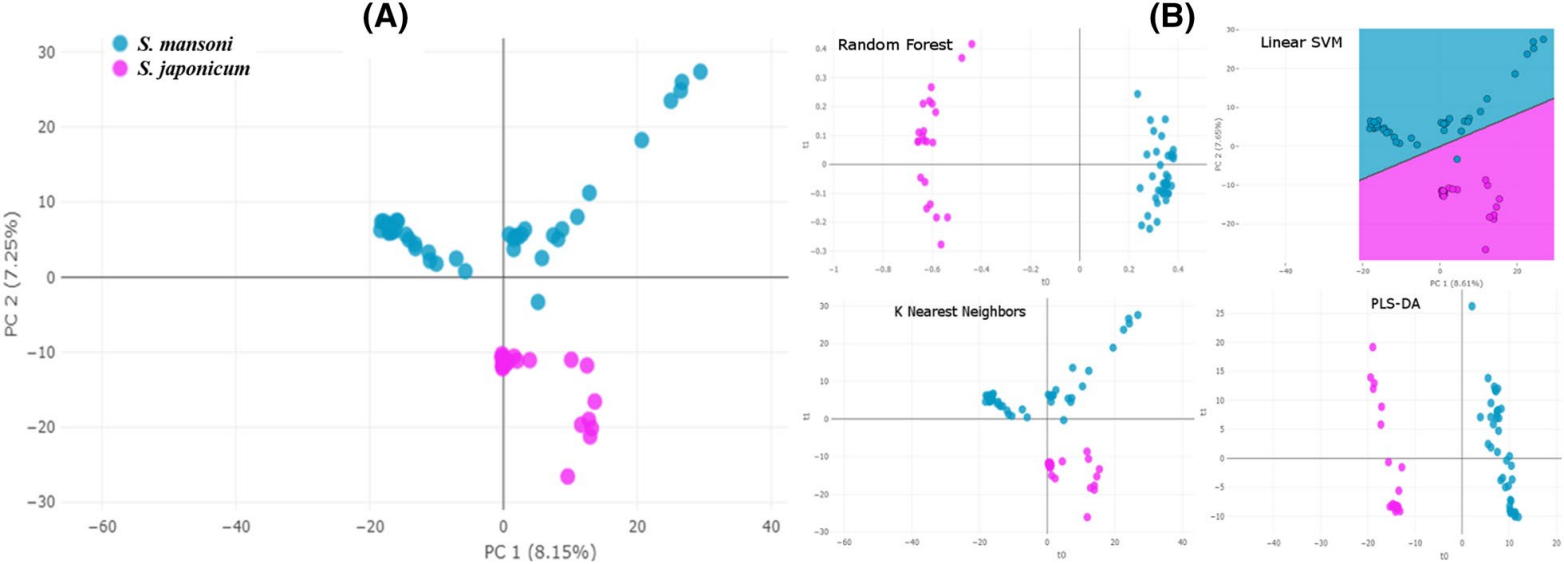
**a**, **b** Interspecies classification of adult worms of *Schistosoma* species examined using unsupervised and supervised algorithms. **a** Two-dimensional view of a principal component (*PC*) analysis (PCA) based on a peak matrix generated with a threshold of 1% and total ion current (TIC) normalization (TICp). **b** Two-dimensional view showing the distance plot of four classification algorithms [Random Forest (RF), linear support vector machine (*SVM*),* k*-nearest neighbors (KNN), and partial least square-discriminant analysis (*PLS-DA*)] using Clover MS Data Analysis software

Peak matching analysis using parameters described in the “Classification using ML algorithms” section generated a peak matrix which was used as input for classification. A PCA based on detected peaks showed clear separation between *S. mansoni* and *S. japonicum* (Fig. 3a). Likewise, all the supervised classification algorithms (SVM, RF, PLS-DA, KNN) showed good discrimination between *S. mansoni* and *S. japonicum* (Fig. 3b). The tenfold cross-validation results indicated accuracy values ranging from 96.8 to 100%, and F1 scores (the harmonic mean of precision and sensitivity) between 95.5% and 100% (Table 2), and thus indicated a species-specific spectra profile. However, the RF algorithm showed better results, with no misclassification, i.e., an accuracy of 100% and an F1 score of 100%. Moreover, area under receiver operating characteristic curve values (0.955 for SVM, 0.995 for PLS-DA, 0.995 for RF, and 0.983 for KNN) and area under the precision-recall curve values (0.98 for SVM, 1.00 for PLS-DA, 1.00 for RF, and 0.97 for KNN) confirmed these observations (Additional file 2: Fig. S1).

**Table 2:** Tenfold cross-validation results showing scores (%) obtained with four different classification algorithms [linear support vector machine (*SVM*), partial least squares-discriminant analysis (*PLS-DA*), Random Forest (*RF*), and* k*-nearest neighbors (*KNN*)]

*Schistosoma mansoni* vs *Schistosoma japonicum*^a^			
Actual group	Predicted group		% Correct
	*S. japonicum*	*S. mansoni*	
SVM			
* S. japonicum*	22 (TP)	0 (FN)	100 (Sensitivity)
* S. mansoni*	2 (FP)	38 (TN)	95 (Specificity)
	91.7% (PPV)	100% (NPV)	96.8 (Accuracy)
			95.7 (F1 score)
PLS-DA			
* S. japonicum*	21 (TP)	1 (FN)	95.5 (Sensitivity)
* S. mansoni*	1 (FP)	39 (TN)	97.5 (Specificity)
	95.5% (PPV)	97.5% (NPV)	96.8 (Accuracy)
			95.5 (F1 score)
RF			
* S. japonicum*	22 (TP)	0 (FN)	100 (Sensitivity)
* S. mansoni*	0 (FP)	40 (TN)	100 (Specificity)
	100% (PPV)	100% (NPV)	100 (Accuracy)
			100 (F1 score)
KNN			
* S. japonicum*	21 (TP)	1 (FN)	95.5 (Sensitivity)
* S. mansoni*	1 (FP)	39 (TN)	97.5 (Specificity)
	95.5% (PPV)	97.5% (NPV)	96.8 (Accuracy)
			95.5 (F1 score)

*S. mansoni* (ethanol vs RNA*later*)^b^			
Actual group	Predicted group		% Correct
	RNA*later*	Ethanol	
SVM			
RNA*later*	20 (TP)	1 (FN)	95.24 (Sensitivity)
Ethanol	0 (FP)	19 (TN)	100 (Specificity)
	100% (PPV)	95% (NPV)	97.5 (Accuracy)
			97.6 (F1 score)
PLS-DA			
RNA*later*	21 (TP)	0 (FN)	100 (Sensitivity)
Ethanol	0 (FP)	19 (TN)	100 (Specificity)
	100% (PPV)	100% (NPV)	100 (Accuracy)
			100 (F1 score)
RF			
RNA*later*	21 (TP)	0 (FN)	100 (Sensitivity)
Ethanol	0 (FP)	19 (TN)	100 (Specificity)
	100% (PPV)	100% (NPV)	100 (Accuracy)
			100 (F1 score)
KNN			
RNA*later*	21 (TP)	0 (FN)	100 (Sensitivity)
Ethanol	0 (FP)	19 (TN)	100 (Specificity)
	100% (PPV)	100% (NPV)	100 (Accuracy)
			100 (F1 score)

*S. japonicum* (ethanol vs RNA*later*)^c^			
Actual group	Predicted group		% Correct
	RNA*later*	Ethanol	
SVM			
RNA*later*	11 (TP)	0 (FN)	100 (Sensitivity)
Ethanol	0 (FP)	11 (TN)	100 (Specificity)
	100% (PPV)	100% (NPV)	100 (Accuracy)
			100 (F1 score)
PLS-DA			
RNA*later*	11 (TP)	0 (FN)	100 (Sensitivity)
Ethanol	0 (FP)	11 (TN)	100 (Specificity)
	100% (PPV)	100% (NPV)	100 (Accuracy)
			100 (F1 score)
RF			
RNA*later*	11 (TP)	0 (FN)	100 (Sensitivity)
Ethanol	0 (FP)	11 (TN)	100 (Specificity)
	100% (PPV)	100% (NPV)	100 (Accuracy)
			100 (F1 score)
KNN			
RNA*later*	11 (TP)	0 (FN)	100 (Sensitivity)
Ethanol	0 (FP)	11 (TN)	100 (Specificity)
	100% (PPV)	100% (NPV)	100 (Accuracy)
			100 (F1 score)

*TP* True positive, *TN* true negative, *FP* false positive, *FN* false negative, *PPV* positive predictive values, *NPV* negative predictive values

^a^Discrimination of adult* S. japonicum* from adult* S. mansoni*;* S. japonicum* considered a positive category

^b^Discrimination of* S. mansoni* isolates based on the effect of the storage medium; RNA*later* considered a positive category

^c^Discrimination of* S. japonicum* isolates based on the effect of the storage medium; RNA*later* considered a positive category

### Intraspecies classification

**Fig. 4:**
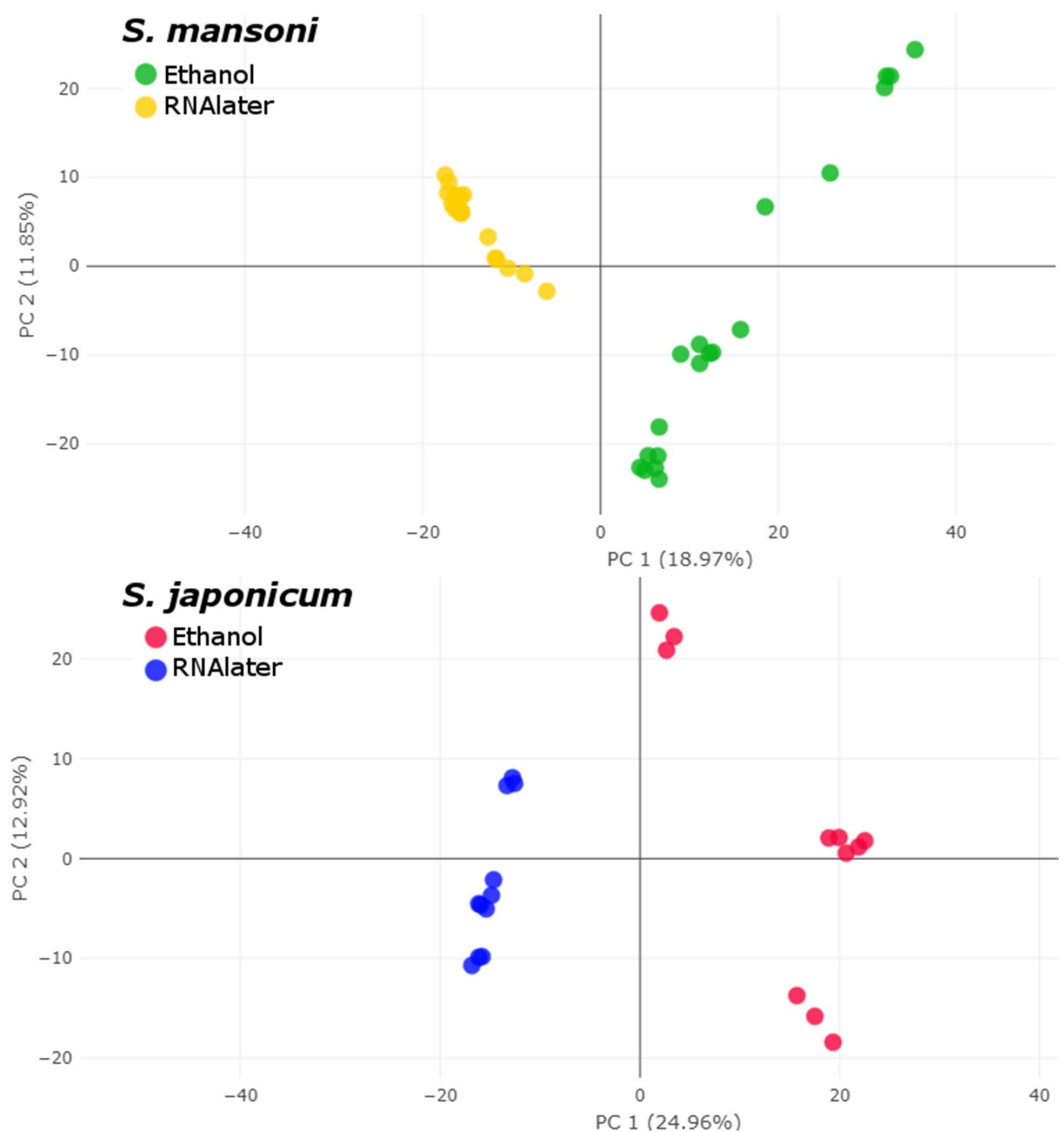
Intraspecies classification of adult worms of *Schistosoma* species by an unsupervised algorithm. Two-dimensional view of a PCA based on a peak matrix generated with a threshold of 1% and TICp. For abbreviations, see Fig. 3

**Fig. 5:**
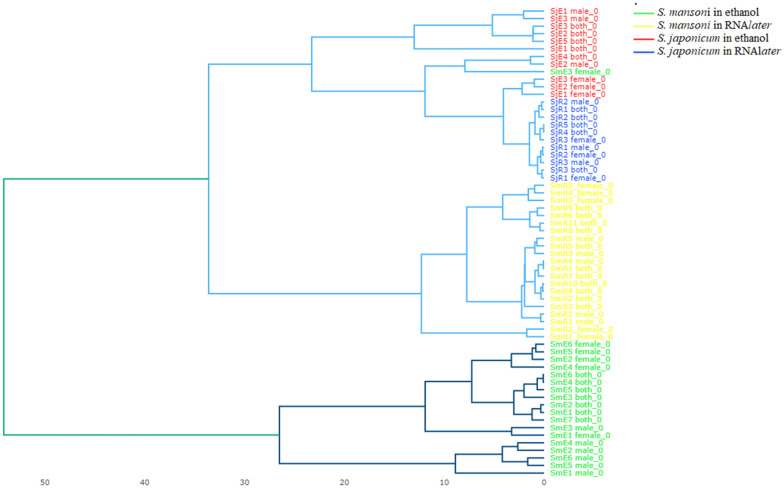
Dendrogram analysis. Hierarchical clustering based on spectral data showing the relatedness of adult *Schistosoma mansoni* and *Schistosoma japonicum* stored in RNA*later* and in ethanol

**Fig. 6:**
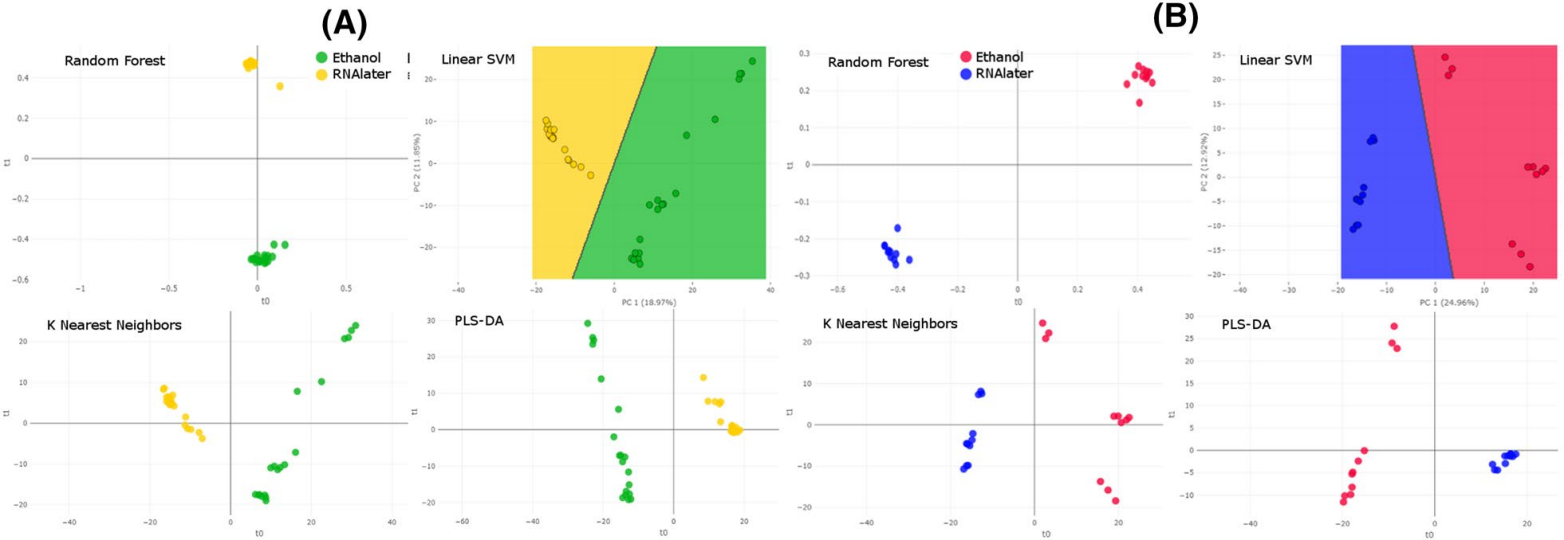
**a**, **b** Intraspecies classification of adult *Schistosoma* by supervised algorithms. Two-dimensional view of the results of four classification algorithms (RF, linear SVM, KNN, and PLS-DA) based on a peak matrix generated with a threshold of 1% and TICp, using Clover MS Data Analysis software. **a** Classification of *Schistosoma mansoni* isolates stored in 70% ethanol versus those stored in RNA*later*. **b** Classification of *Schistosoma japonicum* isolates in 70% ethanol versus those stored in RNA*later*. For abbreviations, see Fig. 3

To further investigate the intraspecies variability of the spectra based on the different storage solutions, the dataset was split into two subsets: one subset with all isolates of *S. mansoni*, and one with *S. japonicum* isolates. Peak matrices generated for each dataset were then used as input for intraspecies classification. For both species, the PCA revealed clear discrimination between isolates stored in 70% (*v*/*v*) and those stored in RNA*later* (Fig. 4)*.* In addition, the dendrogram analysis showed similar results, with distinct groups and with a clear separation between the two species. Moreover, we also observed distinct groups where samples kept in the same storage medium clustered together, except for one sample (*S. mansoni* female 3, in ethanol) that was misclassified (Fig. 5). These observations were confirmed by all the supervised classification algorithms tested, i.e., SVM, PLS-DA, RF, and KNN (Fig. 6a, b). The* k*-fold cross-validation of both datasets showed an accuracy of 100% and an F1 score of 100% for all the tested algorithms, except for SVM tested with the *S. mansoni* dataset (accuracy 97.5%, F1 score 97.6%) (Table 2).

## Discussion

Species-specific spectra generated by MALDI-TOF MS allowed for the identification and specific differentiation of adult *Schistosoma* worms. Indeed, all the isolates were correctly identified, and an LSV of ≥ 2, which is the reference for species-specific identification for bacteria, was achieved for 81% of all the isolates. Of note, no misidentification between the two species was observed. Hence, these results confirm previous reports on the applicability of MALDI-TOF MS for the identification of trematode species [10, 21]. The application of MALDI-TOF MS for parasite identification has attracted considerable attention, and the technique has been employed for other helminth species such as nematodes, i.e., *Trichuris* spp. [22] and *Anisakis* spp. [23], and the cestode *Taenia saginata* [17].

We were able to confirm the robustness of this approach for the reliable differentiation of adult *S. mansoni* from adult *S. japonicum* through classification of the protein spectra using unsupervised (PCA) and supervised (SVM, PLS-DA, RF, and KNN) ML algorithms. The successful application of ML algorithm classification has been repeatedly demonstrated for microbes [19], e.g., for the subtyping of bacterial species [24], and for the detection of antimicrobial resistance [18, 25]. More recently, studies have also been published on the application of ML algorithms for parasite identification. Kalafi et al. [26] reported the automated identification of monogeneans (flatworms present on the gills and skin of fish), with an overall classification accuracy of 90% using KNN, and an accuracy of 91.25% using the leave-one-out cross-validation method. An ML-based study that used nucleotide sequence analysis for the taxonomic evaluation of *Strongyloides fuelleborni* and *Strongyloides stercoralis* also indicated the utility, and another possible application, of this approach [27].

An effect of storage solution on spectra profiles was observed in the present study. Indeed, differences between the spectra profiles of samples stored in ethanol or RNA*later* were noted during a preliminary visual inspection. Furthermore, both unsupervised and supervised algorithms showed that isolates of the same species clustered differently according to the storage solution used. Of note, PCA, SVM, and KNN highlighted sub-clustering based on sex (or type) for samples stored in ethanol, with three subgroups corresponding to males, females, and mixed (male, and female) samples, respectively.

In applied and diagnostic parasitology, a host of different protocols for sample collection and storage are commonly employed. Alcohol or formalin are most frequently utilized as storage solutions, especially for long-term storage outside of a fridge or freezer [8]. Most published studies on the identification of parasites via MALDI-TOF MS used either 70% (*v*/*v*) ethanol [10, 21, 28] or sodium chloride [29] for sample preservation. Wendel et al. [17] recently evaluated the effect of four different storage media [ethanol 70% (*v*/*v*), sodium chloride 0.45% (v/), LC–MS grade water, and formalin 37% (*v*/*v*)] on the identification of *T. saginata* proglottids, and reported similar results in terms of spectra profiles and identification score values for all media for up to 24 weeks of storage, except for formalin 37% (*v*/*v*), for which no spectrum was obtained [17]. Mayer-Scholl et al. [30] evaluated the impact of different conservation conditions (sample freezing, preservation in ethanol 70%) and reported only minor, non-significant, variations in the generated spectra, which did not significantly affect identification. However, in the present study we observed different peaks and different peak intensities depending on the storage solution used. ML algorithms hold promise for the differentiation of samples stored in these different storage media. Furthermore, the accuracy of identification for new, independent samples using the helminth database was better for samples stored in RNA*later* (95% and 100% correct identification at the species level for *S. mansoni* and S*. japonicum*, respectively) than for samples stored in ethanol (correct species identification of 61% for *S. mansoni* and 70% and *S. japonicum*). These results may indicate that preservation in RNA*late*r offers better stability and better integrity of the proteins (Table 2, External validation). Saito et al. [31] demonstrated that RNA*later* was more effective than ethanol in preventing the degradation of proteins from *Synechococcus* WH8102 (a marine cyanobacterium), which were preserved in a total of five different solutions.

This study had several limitations. First, the number of samples was relatively small and originated from animals—experimentally infected mice—kept in just a few different settings. Indeed, before this method is used in laboratories that carry out routine diagnostics, additional research is warranted to validate our approach with a larger number of samples stemming from different locations and various hosts. Second, another limitation was that we did not examine samples of the parasites at different development stages, i.e., we only investigated adult worms, not eggs or larvae. As infected human beings expel eggs through their feces or urine, future studies should try to apply a similar approach to the one used here for eggs originating from stool specimens (in the case of *S. mansoni* and *S. japonicum*) and/or urine samples (in the case of *S. haematobium*). Third, due to a lack of new, independent samples, not all of the ML models used for classification could be subjected to external validation. External validation (a blind test) using new samples is desirable to further validate the performance of the ML models.

## Conclusions

To our knowledge, this is the first study to employ MALDI-TOF MS for the identification of adult *Schistosoma* worms. Our results provide evidence that MALDI-TOF MS is an efficient, rapid, and promising tool for the reliable identification and differentiation of adult *Schistosoma* worms. The creation, and validation, of species-specific reference spectra is necessary in the absence of a commercially available database for parasite identification. Thus, ML-based classification algorithms could also be used as predictive models for parasite species discrimination, as well as for the detection of possible variations in spectra profiles caused by the storage media employed.

## Supplementary Information

**Additional file 1: Table S1.** MALDI identification using the commercial database released by Bruker Daltonics for bacterial identification to check the purity of the spectra and possible contamination with spectra from bacteria.

**Additional file 2: Figure S1. **Receiver operating characteristic (ROC) and precision-recall (PR) curves and their related areas under the curves [area under receiver operating characteristic curve (AUROC) and area under the precision-recall curve (AUPR)] for *Schistosoma* species classification using supervised machine learning (ML) algorithms.** a** Support vector machine, **b** partial least square–discriminant analysis (PLS-DA), **c** Random Forest (RF), and **d*** k*-nearest neighbor (KNN).

## Data Availability

The data presented in this study are available on reasonable request from the corresponding author.

## Abbreviations

*COX1*: Cytochrome oxidase 1 gene
KNN: *k*-nearest neighbor
LC–MS: Liquid chromatography-mass spectrometry
LSV: Log score value
MALDI-TOF MS: Matrix-assisted laser desorption ionization time-of-flight mass spectrometry
ML: Machine learning
MSP: Main spectra profile
PCA: Principal component analysis
PLS-DA: Partial least square-discriminant analysis
RF: Random Forest
SVM: Support vector machine
