# Supplementary material for: Matrix-assisted laser desorption/ionization time-of-flight mass spectrometry for differential identification of adult Schistosoma worms
Source: Parasit Vectors. 2023 Jan 19;16:20. doi: 10.1186/s13071-022-05604-0 (PMC9854196; doi:10.1186/s13071-022-05604-0)
Supplement: Supplementary file 1 — Additional file 1: Table S1. MALDI identification using the commercial database released by Bruker Daltonics for bacterial identification to check the purity of the spectra and possible contamination with spectra from bacteria. [file 13071_2022_5604_MOESM1_ESM.docx]

**Additional file 1: Table S1:** MALDI identification using the commercial database released by Bruker for bacterial identification to check the purity of the spectra and verify possible contamination with spectra from bacteria.

| **Species name** | **Nb. Of spectra** | **MALDI score** |
| --- | --- | --- |
| ***Schistosoma_japonicum_sjE_both_3*** | **22** | **1.38** |
| *Candida krusei ATCC 6258 THL* | 1 | 1.33 |
| *Candida krusei CBS 2457 CBS* | 1 | 1.40 |
| *Candida tropicalis ATCC 13803 THL* | 6 | 1.43 |
| *Corynebacterium bovis DSM 20582T DSM* | 2 | 1.32 |
| *Hydrogenophaga flava B339 UFL* | 2 | 1.36 |
| *Lactobacillus curvatus DSM 20496 DSM* | 4 | 1.39 |
| *Myroides odoratus DSM 2801T HAM* | 3 | 1.39 |
| *Neisseria meningitidis 639 PGM* | 1 | 1.32 |
| *Staphylococcus kloosii DSM 20676T DSM* | 2 | 1.37 |
| ***Schistosoma_japonicum_sjE_both_4*** | **28** | **1.40** |
| *Candida krusei ATCC 14243 THL* | 2 | 1.40 |
| *Candida krusei ATCC 6258 THL* | 3 | 1.38 |
| *Candida krusei CBS 2457 CBS* | 1 | 1.36 |
| *Candida tropicalis ATCC 13803 THL* | 2 | 1.38 |
| *Hydrogenophaga flava B339 UFL* | 11 | 1.46 |
| *Lactobacillus curvatus DSM 20496 DSM* | 3 | 1.38 |
| *Myroides odoratus DSM 2801T HAM* | 2 | 1.37 |
| *Neisseria meningitidis 639 PGM* | 1 | 1.22 |
| *Neisseria meningitidis C1 2 PGM* | 1 | 1.33 |
| *Staphylococcus epidermidis 6b_s ESL* | 1 | 1.35 |
| *Thauera linaloolentis 47Lol MPB* | 1 | 1.36 |
| ***Schistosoma_japonicum_sjE_both_5*** | **29** | **1.35** |
| *Arthrobacter monumenti DSM 16405T DSM* | 1 | 1.39 |
| *Brevundimonas aurantiaca DSM 4731T HAM* | 1 | 1.38 |
| *Candida tropicalis ATCC 13803 THL* | 3 | 1.37 |
| *Corynebacterium bovis DSM 20582T DSM* | 2 | 1.35 |
| *Hydrogenophaga flava B339 UFL* | 7 | 1.37 |
| *Kytococcus sedentarius IMET 11362T HKJ* | 2 | 1.32 |
| *Lactobacillus perolens DSM 12745 DSM* | 2 | 1.36 |
| *Legionella birminghamensis HWL_126 HWH* | 1 | 1.34 |
| *Neisseria meningitidis 639 PGM* | 1 | 1.27 |
| *Neisseria meningitidis C1 2 PGM* | 2 | 1.31 |
| *Paracoccus versutus B352 UFL* | 2 | 1.32 |
| *Pseudarthrobacter oxydans DSM 20119T DSM* | 1 | 1.27 |
| *Staphylococcus condimenti DSM 11675 DSM* | 2 | 1.35 |
| *Staphylococcus kloosii DSM 20676T DSM* | 1 | 1.31 |
| *Streptomyces sp HKI 48 HKJ* | 1 | 1.34 |
| ***Schistosoma_japonicum_sjE_female_1*** | **31** | **1.31** |
| *Actinomyces funkei CCUG 61727 CCUG* | 1 | 1.38 |
| *Aeromonas sobria CECT 4245T DSM* | 1 | 1.26 |
| *Arthrobacter citreus IMET 10680T HKJ* | 2 | 1.29 |
| *Brevundimonas nasdae DSM 14572T HAM* | 1 | 1.30 |
| *Candida boidinii DSM 70034 DSM* | 2 | 1.28 |
| *Candida tropicalis ATCC 13803 THL* | 1 | 1.38 |
| *Candida tropicalis CBS 2314 CBS* | 3 | 1.33 |
| *Corynebacterium flavescens IMET 11080T HKJ* | 1 | 1.29 |
| *Lactobacillus satsumensis DSM 16230T DSM* | 1 | 1.28 |
| *Moraxella catarrhalis MB_8374_05 THL* | 1 | 1.31 |
| *Neisseria gonorrhoeae ATCC 49226 THL* | 1 | 1.30 |
| *Neisseria meningitidis C1 2 PGM* | 3 | 1.33 |
| *Prevotella bivia HU51442_4 PNU* | 2 | 1.28 |
| *Shewanella algae DSM 9167T HAM* | 1 | 1.32 |
| *Staphylococcus haemolyticus 10024 CHB* | 1 | 1.28 |
| *Staphylococcus haemolyticus Mb18803_2 CHB* | 6 | 1.36 |
| *Staphylococcus sciuri ssp sciuri DSM 6671 DSM* | 1 | 1.28 |
| *Stenotrophomonas maltophilia (Pseudomonas beteli) LMG 978T HAM* | 1 | 1.15 |
| *Trueperella bialowiezensis DSM 17162T DSM* | 1 | 1.18 |
| ***Schistosoma_japonicum_sjE_female_2*** | **30** | **1.30** |
| *Acinetobacter baumannii 13101_1 CHB* | 1 | 1.30 |
| *Aeromonas veronii CECT 4199T DSM* | 1 | 1.37 |
| *Arthrobacter globiformis DSM 20124T DSM* | 1 | 1.26 |
| *Bulleidia extructa P10bue_li3AN USH* | 2 | 1.32 |
| *Candida tropicalis ATCC 13803 THL* | 2 | 1.36 |
| *Corynebacterium bovis DSM 20582T DSM* | 4 | 1.34 |
| *Moraxella catarrhalis MB_8374_05 THL* | 4 | 1.33 |
| *Neisseria gonorrhoeae ATCC 49226 THL* | 3 | 1.29 |
| *Neisseria meningitidis C1 2 PGM* | 5 | 1.30 |
| *Pantoea agglomerans CCM 4412 CCM* | 1 | 1.28 |
| *Paracoccus yeei 08526023 MCU* | 1 | 1.17 |
| *Shewanella algae DSM 9167T HAM* | 2 | 1.25 |
| *Staphylococcus haemolyticus Mb18803_2 CHB* | 2 | 1.27 |
| *Weissella halotolerans DSM 20190T DSM* | 1 | 1.29 |
| ***Schistosoma_japonicum_sjE_female_3*** | **30** | **1.36** |
| *Arthrobacter citreus DSM 20133T DSM* | 1 | 1.34 |
| *Arthrobacter citreus IMET 10680T HKJ* | 1 | 1.31 |
| *Candida tropicalis ATCC 13803 THL* | 6 | 1.38 |
| *Candida tropicalis CBS 2314 CBS* | 5 | 1.37 |
| *Corynebacterium flavescens IMET 11080T HKJ* | 1 | 1.26 |
| *Moraxella catarrhalis MB_8374_05 THL* | 2 | 1.37 |
| *Neisseria gonorrhoeae ATCC 49226 THL* | 2 | 1.36 |
| *Staphylococcus haemolyticus 10024 CHB* | 7 | 1.37 |
| *Staphylococcus haemolyticus Mb18803_2 CHB* | 5 | 1.38 |
| ***Schistosoma_japonicum_sjE_male_1*** | **32** | **1.39** |
| *Agromyces bracchium HKI 303 DSM 14596T HKJ* | 1 | 1.31 |
| *Enterococcus faecium VRE_PX_16086218 MLD* | 1 | 1.38 |
| *Filifactor villosus 1051_NCTC 11220T BOG* | 1 | 1.43 |
| *Hydrogenophaga flava B339 UFL* | 6 | 1.39 |
| *Lactobacillus equi DSM 15833T DSM* | 15 | 1.42 |
| *Lactobacillus fuchuensis DSM 14341 DSM* | 1 | 1.38 |
| *Lactobacillus reuteri DSM 20053 DSM* | 1 | 1.36 |
| *Paracoccus versutus B352 UFL* | 1 | 1.31 |
| *Staphylococcus haemolyticus 10024 CHB* | 2 | 1.33 |
| *Staphylococcus kloosii DSM 20676T DSM* | 3 | 1.35 |
| ***Schistosoma_japonicum_sjE_male_2*** | **27** | **1.32** |
| *Blastomonas ursincola DSM 9006T HAM* | 3 | 1.32 |
| *Candida dubliniensis SA 121 CBS* | 1 | 1.26 |
| *Candida guilliermondii RV490_Feb09_02 PSB* | 1 | 1.24 |
| *Filifactor villosus 1051_NCTC 11220T BOG* | 1 | 1.38 |
| *Lactobacillus equi DSM 15833T DSM* | 6 | 1.31 |
| *Neisseria meningitidis C1 2 PGM* | 4 | 1.30 |
| *Pseudomonas poae DSM 14936T HAM* | 2 | 1.37 |
| *Staphylococcus kloosii DSM 20676T DSM* | 9 | 1.33 |
| ***Schistosoma_japonicum_sjE_male_3*** | **24** | **1.39** |
| *Bacillus circulans DSM 11T DSM* | 1 | 1.29 |
| *Candida krusei ATCC 6258 THL* | 11 | 1.39 |
| *Chryseobacterium joostei LMG 18212T HAM* | 1 | 1.36 |
| *Enterococcus faecium VRE_PX_16086218 MLD* | 2 | 1.41 |
| *Jeotgalibacillus marinus DSM 1297T DSM* | 1 | 1.38 |
| *Lactobacillus fuchuensis DSM 14340T DSM* | 1 | 1.37 |
| *Paracoccus versutus B352 UFL* | 4 | 1.41 |
| *Pichia occidentalis CBS 1910 CBS* | 1 | 1.39 |
| *Staphylococcus haemolyticus 10024 CHB* | 1 | 1.52 |
| *Staphylococcus kloosii DSM 20676T DSM* | 1 | 1.37 |
| ***Schistosoma_japonicum_sjR_both_3*** | **26** | **1.29** |
| *Burkholderia vietnamiensis LMG 10929T HAM* | 1 | 1.27 |
| *Candida tropicalis ATCC 13803 THL* | 1 | 1.26 |
| *Clostridium novyi A 1025_NCTC 538 BOG* | 2 | 1.30 |
| *Clostridium ramosum CCUG 45030 CCUG* | 2 | 1.30 |
| *Corynebacterium confusum DSM 44384T DSM* | 2 | 1.24 |
| *Hathewaya histolytica 1036_NCTC 503T BOG* | 2 | 1.27 |
| *Janthinobacterium lividum CIP 106720T HAM* | 2 | 1.31 |
| *Lactobacillus graminis DSM 20719T DSM* | 3 | 1.33 |
| *Lactobacillus sakei ssp sakei DSM 20017T DSM* | 1 | 1.31 |
| *Methylobacterium rhodesianum MB94 UFL* | 1 | 1.22 |
| *Rhizobium radiobacter B166 UFL* | 1 | 1.27 |
| *Streptococcus dysgalactiae ssp dysgalactiae DSM 20662T DSM* | 1 | 1.32 |
| *Streptococcus uberis CCUG 27630 CCUG* | 2 | 1.27 |
| *Streptomyces albus B262 UFL* | 1 | 1.38 |
| *Trichosporon asahii CBS 2530 CBS* | 4 | 1.28 |
| ***Schistosoma_japonicum_sjR_both_4*** | **31** | **1.29** |
| *Acidovorax delafieldii DSM 64T HAM* | 2 | 1.27 |
| *Burkholderia cepacia_Group 18875_1 CHB* | 4 | 1.26 |
| *Burkholderia multivorans LMG 14293 HAM* | 1 | 1.30 |
| *Candida tropicalis ATCC 13803 THL* | 4 | 1.33 |
| *Clostridium ramosum CCUG 45030 CCUG* | 3 | 1.33 |
| *Janthinobacterium lividum CIP 106720T HAM* | 3 | 1.29 |
| *Methylobacterium rhodesianum MB94 UFL* | 1 | 1.20 |
| *Pseudarthrobacter oxydans IMET 10684T HKJ* | 4 | 1.32 |
| *Staphylococcus pasteuri CCUG 48128 CCUG* | 1 | 1.25 |
| *Streptomyces albus B262 UFL* | 3 | 1.31 |
| *Trichosporon asahii CBS 2530 CBS* | 5 | 1.27 |
| ***Schistosoma_japonicum_sjR_both_5*** | **27** | **1.27** |
| *Acidovorax delafieldii DSM 64T HAM* | 1 | 1.24 |
| *Bacillus gibsonii DSM 8722T DSM* | 1 | 1.24 |
| *Burkholderia multivorans LMG 14293 HAM* | 2 | 1.30 |
| *Clostridium novyi A 1025_NCTC 538 BOG* | 1 | 1.25 |
| *Clostridium ramosum CCUG 45030 CCUG* | 2 | 1.31 |
| *Corynebacterium flavescens IMET 11080T HKJ* | 2 | 1.29 |
| *Janthinobacterium lividum CIP 106720T HAM* | 3 | 1.28 |
| *Lactobacillus intestinalis DSM 6629T DSM* | 3 | 1.28 |
| *Methylobacterium rhodesianum MB94 UFL* | 3 | 1.28 |
| *Penicillium nalgiovense 411 UGB* | 1 | 1.25 |
| *Pseudarthrobacter oxydans IMET 10684T HKJ* | 1 | 1.28 |
| *Streptococcus uberis CCUG 27630 CCUG* | 1 | 1.26 |
| *Trichosporon asahii CBS 2530 CBS* | 2 | 1.23 |
| *Trichosporon asahii CBS 7632_C CBS* | 4 | 1.28 |
| ***Schistosoma_japonicum_sjR_female_1*** | **31** | **1.25** |
| *Agromyces cerinus ssp nitratus HKI 11532_DSM 8596T HKJ* | 1 | 1.19 |
| *Burkholderia multivorans LMG 14293 HAM* | 3 | 1.25 |
| *Candida intermedia MY_RV4_12 ERL* | 1 | 1.19 |
| *Candida parapsilosis ATCC 22019 THL* | 1 | 1.18 |
| *Candida tropicalis ATCC 13803 THL* | 2 | 1.25 |
| *Candida tropicalis CBS 4913 CBS* | 1 | 1.23 |
| *Clostridium ramosum 1C15007811_8 MVD* | 1 | 1.26 |
| *Corynebacterium bovis DSM 20582T DSM* | 1 | 1.21 |
| *Flavobacterium saccharophilum DSM 1811T HAM* | 1 | 1.23 |
| *Lactobacillus kefiri DSM 20588 DSM* | 2 | 1.27 |
| *Methylobacterium rhodesianum MB94 UFL* | 1 | 1.20 |
| *Paracoccus versutus B352 UFL* | 2 | 1.28 |
| *Rhizobium radiobacter B166 UFL* | 1 | 1.22 |
| *Scedosporium aurantiacum 149 VML* | 1 | 1.26 |
| *Staphylococcus haemolyticus Mb18803_2 CHB* | 8 | 1.27 |
| *Streptococcus oralis DSM 20627T DSM* | 2 | 1.28 |
| *Trichosporon asahii CBS 4829 CBS* | 1 | 1.21 |
| *Vibrio vulnificus CCUG 38429 CCUG* | 1 | 1.29 |
| ***Schistosoma_japonicum_sjR_female_2*** | **27** | **1.28** |
| *Burkholderia multivorans LMG 14293 HAM* | 1 | 1.22 |
| *Campylobacter coli 11167_03 NVU* | 1 | 1.22 |
| *Candida krusei ATCC 14243 THL* | 3 | 1.26 |
| *Candida krusei CBS 2107 CBS* | 1 | 1.28 |
| *Clostridium ramosum 1C15007811_8 MVD* | 1 | 1.30 |
| *Delftia acidovorans DSM 39T HAM* | 1 | 1.35 |
| *Hanseniaspora uvarum DSM 70788 DSM* | 1 | 1.31 |
| *Lactobacillus reuteri DSM 20056 DSM* | 1 | 1.26 |
| *Paracoccus versutus B352 UFL* | 5 | 1.32 |
| *Penicillium citrinum 1376 MPA* | 1 | 1.26 |
| *Staphylococcus haemolyticus Mb18803_2 CHB* | 1 | 1.31 |
| *Staphylococcus intermedius P_9B JUT* | 1 | 1.26 |
| *Stenotrophomonas acidaminiphila DSM 13117T HAM* | 1 | 1.20 |
| *Streptococcus cristatus DSM 8249T DSM* | 1 | 1.29 |
| *Streptococcus oralis NRZ 37307 NRZ* | 2 | 1.30 |
| *Streptococcus oralis NRZ 38252 NRZ* | 1 | 1.20 |
| *Thauera phenylacetica B4P MPB* | 1 | 1.37 |
| *Trichosporon asahii CBS 2530 CBS* | 1 | 1.27 |
| *Trichosporon asahii CBS 4829 CBS* | 2 | 1.27 |
| ***Schistosoma_japonicum_sjR_female_3*** | **31** | **1.27** |
| *Burkholderia multivorans LMG 14293 HAM* | 2 | 1.30 |
| *Campylobacter hyointestinalis CCUG 14169T NVU* | 1 | 1.30 |
| *Candida dubliniensis CBS 8501 CBS* | 1 | 1.31 |
| *Candida krusei ATCC 14243 THL* | 8 | 1.34 |
| *Candida krusei CBS 2107 CBS* | 4 | 1.27 |
| *Candida parapsilosis ATCC 22019 THL* | 1 | 1.25 |
| *Candida parapsilosis DSM 70125 DSM* | 1 | 1.17 |
| *Candida tropicalis ATCC 13803 THL* | 2 | 1.32 |
| *Lactobacillus graminis DSM 20719T DSM* | 1 | 1.21 |
| *Penicillium citrinum DSM 1997 DSM* | 1 | 1.24 |
| *Rhizobium radiobacter B166 UFL* | 1 | 1.19 |
| *Streptococcus oralis DSM 20627T DSM* | 2 | 1.19 |
| *Streptococcus oralis NRZ 32423 NRZ* | 1 | 1.29 |
| *Streptococcus oralis NRZ 37174 NRZ* | 2 | 1.26 |
| *Trichosporon asahii CBS 2530 CBS* | 1 | 1.26 |
| *Trichosporon asahii CBS 4829 CBS* | 1 | 1.25 |
| *Vibrio diazotrophicus VN_03198 AWIH* | 1 | 1.16 |
| ***Schistosoma_japonicum_sjR_male_1*** | **31** | **1.33** |
| *Bacillus odysseyi DSM 18869T DSM* | 1 | 1.30 |
| *Candida tropicalis ATCC 13803 THL* | 1 | 1.25 |
| *Clostridium novyi A 1025_NCTC 538 BOG* | 5 | 1.30 |
| *Clostridium ramosum CCUG 45030 CCUG* | 2 | 1.32 |
| *Enterococcus avium LMG 16310 LMG* | 2 | 1.32 |
| *Hathewaya histolytica 1036_NCTC 503T BOG* | 1 | 1.26 |
| *Janthinobacterium lividum CIP 106720T HAM* | 15 | 1.37 |
| *Paraburkholderia caribensis DSM 13236T HAM* | 1 | 1.30 |
| *Rhizobium rubi DSM 6772T HAM* | 2 | 1.34 |
| *Trichosporon asahii CBS 2530 CBS* | 1 | 1.23 |
| ***Schistosoma_japonicum_sjR_male_2*** | **30** | **1.31** |
| *Burkholderia cepacia_Group 18875_1 CHB* | 15 | 1.31 |
| *Corynebacterium confusum 287 RLT* | 1 | 1.31 |
| *Corynebacterium confusum DSM 44384T DSM* | 1 | 1.25 |
| *Hanseniaspora uvarum CBS 314T CBS* | 1 | 1.28 |
| *Hathewaya histolytica 1036_NCTC 503T BOG* | 1 | 1.25 |
| *Janthinobacterium lividum CIP 106720T HAM* | 5 | 1.32 |
| *Lactobacillus intestinalis DSM 6629T DSM* | 1 | 1.34 |
| *Staphylococcus hominis ssp novobiosepticus DSM 15614T DSM* | 5 | 1.32 |
| ***Schistosoma_japonicum_sjR_male_3*** | **29** | **1.37** |
| *Burkholderia cepacia_Group 18875_1 CHB* | 4 | 1.32 |
| *Clostridium novyi A 1025_NCTC 538 BOG* | 1 | 1.32 |
| *Hathewaya histolytica 1036_NCTC 503T BOG* | 1 | 1.37 |
| *Janthinobacterium lividum CIP 106720T HAM* | 16 | 1.40 |
| *Lactobacillus intestinalis DSM 6629T DSM* | 1 | 1.28 |
| *Rhizobium rubi DSM 6772T HAM* | 3 | 1.32 |
| *Staphylococcus hominis ssp novobiosepticus DSM 15614T DSM* | 2 | 1.37 |
| *Trichosporon asahii CBS 7632_C CBS* | 1 | 1.30 |
| ***Schistosoma_japonicum_sjE_both_1*** | **27** | **1.39** |
| *Candida tropicalis ATCC 13803 THL* | 8 | 1.39 |
| *Citrobacter braakii 9314_2 CHB* | 1 | 1.40 |
| *Corynebacterium bovis DSM 20582T DSM* | 1 | 1.41 |
| *Hydrogenophaga flava B339 UFL* | 4 | 1.42 |
| *Lactobacillus curvatus DSM 20496 DSM* | 5 | 1.39 |
| *Lactobacillus harbinensis DSM 16991T DSM* | 2 | 1.40 |
| *Lactobacillus perolens DSM 12745 DSM* | 1 | 1.36 |
| *Myroides odoratus DSM 2801T HAM* | 1 | 1.43 |
| *Staphylococcus kloosii DSM 20676T DSM* | 2 | 1.42 |
| *Weissella halotolerans DSM 20190T DSM* | 2 | 1.36 |
| ***Schistosoma_japonicum_sjE_both_2*** | **29** | **1.38** |
| *Campylobacter fetus ssp fetus RV412_A1_2010_02 LBK* | 1 | 1.38 |
| *Candida krusei ATCC 6258 THL* | 3 | 1.38 |
| *Candida tropicalis ATCC 13803 THL* | 3 | 1.38 |
| *Candida utilis DSM 2361 DSM* | 2 | 1.44 |
| *Cryptococcus neoformans ATCC 14116 THL* | 1 | 1.32 |
| *Curtobacterium albidum HKI 11500 HKJ* | 1 | 1.37 |
| *Hydrogenophaga flava B339 UFL* | 5 | 1.37 |
| *Lactobacillus curvatus DSM 20496 DSM* | 6 | 1.40 |
| *Lactobacillus harbinensis DSM 16991T DSM* | 1 | 1.39 |
| *Myroides odoratus DSM 2801T HAM* | 1 | 1.35 |
| *Neisseria meningitidis 639 PGM* | 1 | 1.29 |
| *Staphylococcus kloosii DSM 20676T DSM* | 3 | 1.41 |
| *Weissella halotolerans DSM 20190T DSM* | 1 | 1.39 |
| ***Schistosoma_japonicum_sjR_both_1*** | **32** | **1.32** |
| *Burkholderia cepacia_Group 18875_1 CHB* | 3 | 1.27 |
| *Burkholderia multivorans LMG 14293 HAM* | 2 | 1.31 |
| *Candida krusei ATCC 14243 THL* | 2 | 1.34 |
| *Candida tropicalis ATCC 13803 THL* | 15 | 1.35 |
| *Clostridium novyi A 1025_NCTC 538 BOG* | 1 | 1.29 |
| *Fictibacillus arsenicus 4_2 TUB* | 1 | 1.30 |
| *Listeria seeligeri CCUG 45639 CCUG* | 1 | 1.26 |
| *Novosphingobium aromaticivorans DSM 12444T HAM* | 3 | 1.29 |
| *Staphylococcus pasteuri DSM 10657 DSM* | 2 | 1.27 |
| *Streptococcus dysgalactiae ssp dysgalactiae DSM 20662T DSM* | 2 | 1.26 |
| ***Schistosoma_japonicum_sjR_both_2*** | **27** | **1.30** |
| *Actinomyces graevenitzii P4bue_re2AN USH* | 2 | 1.29 |
| *Aspergillus tritici F75 RLH* | 1 | 1.25 |
| *Bacillus hemicellulosilyticus DSM 16731T DSM* | 1 | 1.29 |
| *Clostridium novyi A 1025_NCTC 538 BOG* | 2 | 1.30 |
| *Corynebacterium flavescens IMET 11080T HKJ* | 1 | 1.33 |
| *Cryptococcus neoformans ATCC 14116 THL* | 2 | 1.24 |
| *Hathewaya histolytica 1036_NCTC 503T BOG* | 6 | 1.31 |
| *Janthinobacterium lividum CIP 106720T HAM* | 2 | 1.34 |
| *Lactobacillus bifermentans DSM 20003T DSM* | 1 | 1.32 |
| *Lactobacillus sakei ssp sakei DSM 20017T DSM* | 1 | 1.29 |
| *Methylobacterium rhodesianum MB99 UFL* | 1 | 1.32 |
| *Rhizobium rubi DSM 6772T HAM* | 5 | 1.28 |
| *Streptomyces violaceoruber B263 UFL* | 1 | 1.30 |
| *Trichosporon asahii CBS 7632_C CBS* | 1 | 1.26 |
| ***Schistosoma_mansoni_female_3*** | **23** | **1.34** |
| *Acidovorax defluvii DSM 12644T HAM* | 1 | 1.39 |
| *Candida tropicalis ATCC 13803 THL* | 1 | 1.32 |
| *Carnobacterium maltaromaticum CIP 102035 CIP* | 1 | 1.42 |
| *Hydrogenophaga flava B339 UFL* | 5 | 1.36 |
| *Kandleria vitulina DSM 20405T DSM* | 2 | 1.40 |
| *Lactobacillus kitasatonis DSM 16761T DSM* | 1 | 1.36 |
| *Lactobacillus perolens DSM 12745 DSM* | 1 | 1.33 |
| *Lactobacillus satsumensis DSM 16230T DSM* | 1 | 1.36 |
| *Lactobacillus vini DSM 20605T DSM* | 1 | 1.29 |
| *Neisseria meningitidis Serogroup_X BRL* | 1 | 1.24 |
| *Pichia occidentalis CBS 1910 CBS* | 1 | 1.31 |
| *Pseudomonas oryzihabitans DSM 6835T HAM* | 4 | 1.32 |
| *Pseudomonas rhizosphaerae LMG 21640T HAM* | 1 | 1.34 |
| *Staphylococcus lugdunensis DSM 4805 DSM* | 1 | 1.29 |
| *Weissella minor DSM 20014T DSM* | 1 | 1.41 |
| ***Schistosoma_mansoni_male_3*** | **32** | **1.37** |
| *Arthrobacter ramosus IMET 10685T HKJ* | 1 | 1.40 |
| *Candida guilliermondii CBS 566 CBS* | 3 | 1.39 |
| *Flavobacterium frigidarium DSM 17623T DSM* | 1 | 1.36 |
| *Hydrogenophaga flava B339 UFL* | 4 | 1.40 |
| *Lactobacillus paralimentarius DSM 13238T DSM* | 2 | 1.33 |
| *Lactobacillus perolens DSM 12745 DSM* | 1 | 1.35 |
| *Paeniclostridium sordellii 1070_ATCC 9714T BOG* | 5 | 1.42 |
| *Pantoea agglomerans CCM 298 CCM* | 1 | 1.30 |
| *Paracoccus versutus B352 UFL* | 1 | 1.39 |
| *Pseudomonas nitroreducens LMG 20221T HAM* | 1 | 1.36 |
| *Pseudomonas rhizosphaerae LMG 21640T HAM* | 2 | 1.30 |
| *Shewanella frigidimarina DSM 12253T HAM* | 7 | 1.34 |
| *Staphylococcus capitis ssp urealyticus DSM 6717T DSM* | 1 | 1.40 |
| *Staphylococcus epidermidis 4b_r ESL* | 1 | 1.34 |
| *Trichosporon loubieri CBS 8265 CBS* | 1 | 1.33 |
| ***Schistosoma_mansoni_male+female_1*** | **26** | **1.29** |
| *Actinomyces odontolyticus CCUG 18309 CCUG* | 1 | 1.22 |
| *Aeromonas caviae 60 PIM* | 1 | 1.32 |
| *Aeromonas jandaei CECT 4228T DSM* | 1 | 1.31 |
| *Aeromonas veronii CECT 4199T DSM* | 1 | 1.25 |
| *Aromatoleum toluolicum T MPB* | 2 | 1.29 |
| *Candida tropicalis ATCC 13803 THL* | 1 | 1.28 |
| *Enterococcus faecium 11037 CHB* | 1 | 1.35 |
| *Helcococcus kunzii CCUG 50466 CCUG* | 1 | 1.32 |
| *Kandleria vitulina DSM 20405T DSM* | 2 | 1.27 |
| *Kytococcus sedentarius IMET 11362T HKJ* | 1 | 1.29 |
| *Lactobacillus crispatus DSM 20356 DSM* | 1 | 1.31 |
| *Lactobacillus delbrueckii ssp lactis DSM 20072T DSM* | 1 | 1.23 |
| *Lactobacillus delbrueckii ssp lactis DSM 20073 DSM* | 3 | 1.36 |
| *Lactobacillus delbrueckii ssp lactis DSM 20076 DSM* | 1 | 1.34 |
| *Lactobacillus mucosae DSM 13345T DSM* | 1 | 1.41 |
| *Luteimonas aestuarii DSM 19680T DSM* | 1 | 1.22 |
| *Neisseria meningitidis 639 PGM* | 1 | 1.27 |
| *Pseudomonas putida B401 UFL* | 1 | 1.18 |
| *Serratia marcescens 13103_1 CHB* | 1 | 1.23 |
| *Streptococcus ovis DSM 16829T DSM* | 2 | 1.29 |
| *Streptomyces lavendulae B264 UFL* | 1 | 1.28 |
| ***Schistosoma_mansoni_male+female_2*** | **21** | **1.32** |
| *Aromatoleum toluolicum T MPB* | 1 | 1.27 |
| *Arthrobacter roseus DSM 14508T DSM* | 1 | 1.24 |
| *Clostridium ramosum CCUG 45030 CCUG* | 1 | 1.29 |
| *Enterobacter bugandensis DSM 101091 DSM* | 1 | 1.16 |
| *Enterococcus faecium PX_21086109_III MLD* | 1 | 1.31 |
| *Hydrogenophaga flava B339 UFL* | 1 | 1.30 |
| *Kandleria vitulina DSM 20405T DSM* | 1 | 1.31 |
| *Lactobacillus acidophilus DSM 20242 DSM* | 1 | 1.34 |
| *Lactobacillus mucosae DSM 13345T DSM* | 4 | 1.37 |
| *Neisseria meningitidis C1 2 PGM* | 6 | 1.34 |
| *Paraburkholderia fungorum LMG 20227T HAM* | 1 | 1.27 |
| *Pichia occidentalis CBS 1910 CBS* | 1 | 1.40 |
| *Pseudomonas putida B401 UFL* | 1 | 1.33 |
| ***Schistosoma_mansoni_male+female_3*** | **31** | **1.28** |
| *Aquabacterium commune DSM 11901T DSM* | 1 | 1.19 |
| *Clostridium tetani 1089_ATCC 10779 BOG* | 3 | 1.30 |
| *Enterococcus faecium PX_21086109_III MLD* | 1 | 1.20 |
| *Hydrogenophaga flava B339 UFL* | 2 | 1.28 |
| *Lactobacillus crispatus DSM 20356 DSM* | 3 | 1.34 |
| *Lactobacillus mucosae DSM 13345T DSM* | 3 | 1.34 |
| *Lactobacillus paracasei ssp paracasei DSM 8742 DSM* | 1 | 1.21 |
| *Lactobacillus sakei ssp sakei DSM 20017T DSM* | 1 | 1.25 |
| *Methylobacterium rhodesianum MB99 UFL* | 2 | 1.24 |
| *Neisseria meningitidis C1 2 PGM* | 7 | 1.32 |
| *Paraburkholderia fungorum LMG 20227T HAM* | 2 | 1.27 |
| *Pseudomonas oryzihabitans DSM 6835T HAM* | 1 | 1.28 |
| *Pseudomonas putida B342T UFL* | 1 | 1.18 |
| *Pseudomonas putida B401 UFL* | 1 | 1.34 |
| *Staphylococcus epidermidis ATCC 12228 CHB* | 1 | 1.26 |
| *Staphylococcus lugdunensis 20659_1 CHB* | 1 | 1.16 |
| ***Schistosoma_mansoni_male+female_4*** | **29** | **1.29** |
| *Aromatoleum toluolicum T MPB* | 2 | 1.36 |
| *Candida lambica CBS 603 CBS* | 1 | 1.28 |
| *Enterococcus faecium PX_21086109_III MLD* | 4 | 1.33 |
| *Kandleria vitulina DSM 20405T DSM* | 2 | 1.32 |
| *Lactobacillus delbrueckii ssp indicus DSM 15996T DSM* | 1 | 1.20 |
| *Lactobacillus delbrueckii ssp lactis DSM 20355 DSM* | 1 | 1.21 |
| *Lactobacillus mucosae DSM 13345T DSM* | 1 | 1.27 |
| *Lactobacillus parabuchneri DSM 5707T DSM* | 1 | 1.26 |
| *Lactobacillus satsumensis DSM 16230T DSM* | 1 | 1.23 |
| *Leptotrichia trevisanii ENR_0477 ENR* | 1 | 1.21 |
| *Neisseria meningitidis C1 2 PGM* | 8 | 1.30 |
| *Pseudomonas alcaligenes DSM 50342T HAM* | 1 | 1.30 |
| *Pseudomonas putida DSM 50198 HAM* | 1 | 1.17 |
| *Serratia marcescens 13103_1 CHB* | 2 | 1.34 |
| *Staphylococcus capitis ssp capitis DSM 20326T DSM* | 1 | 1.17 |
| *Staphylococcus warneri Mb18796_1 CHB* | 1 | 1.26 |
| ***Schistosoma_mansoni_male+female_5*** | **29** | **1.32** |
| *Aromatoleum tolulyticus Tol4 MPB* | 1 | 1.31 |
| *Aromatoleum toluolicum T MPB* | 2 | 1.29 |
| *Enterococcus faecium PX_21086109_III MLD* | 4 | 1.33 |
| *Janthinobacterium lividum CIP 106720T HAM* | 1 | 1.24 |
| *Neisseria meningitidis C1 2 PGM* | 9 | 1.34 |
| *Pseudomonas putida B409 UFL* | 4 | 1.31 |
| *Saccharopolyspora erythraea HKI 184 HKJ* | 2 | 1.38 |
| *Serratia marcescens 13103_1 CHB* | 1 | 1.30 |
| *Sphingomonas paucimobilis B341 UFL* | 1 | 1.38 |
| *Staphylococcus lugdunensis DSM 4805 DSM* | 2 | 1.25 |
| *Stenotrophomonas acidaminiphila DSM 13117T HAM* | 1 | 1.28 |
| *Weeksella virosa LMG 12995T HAM* | 1 | 1.30 |
| ***Schistosoma_mansoni_male+female_6*** | **24** | **1.33** |
| *Aromatoleum evansii KB740 MPB* | 1 | 1.26 |
| *Burkholderia ambifaria LMG 11351 HAM* | 1 | 1.39 |
| *Cryptococcus neoformans ATCC 14116 THL* | 1 | 1.33 |
| *Escherichia coli DH5alpha BRL* | 1 | 1.27 |
| *Escherichia coli ESBL_EA_RSS_1528T CHB* | 1 | 1.21 |
| *Kandleria vitulina DSM 20405T DSM* | 1 | 1.28 |
| *Lactobacillus delbrueckii ssp lactis DSM 20073 DSM* | 1 | 1.25 |
| *Lactobacillus mucosae DSM 13345T DSM* | 1 | 1.25 |
| *Lactobacillus paralimentarius DSM 13961 DSM* | 1 | 1.32 |
| *Neisseria meningitidis C1 2 PGM* | 4 | 1.39 |
| *Pseudomonas putida B401 UFL* | 2 | 1.41 |
| *Pseudomonas putida B409 UFL* | 1 | 1.34 |
| *Serratia marcescens 13103_1 CHB* | 3 | 1.34 |
| *Staphylococcus lugdunensis DSM 4805 DSM* | 3 | 1.35 |
| *Streptomyces lavendulae B264 UFL* | 2 | 1.27 |
| ***Schistosoma_mansoni_male+female_7*** | **27** | **1.29** |
| *Achromobacter spanius LMG 5911T HAM* | 2 | 1.34 |
| *Aeromonas caviae 60 PIM* | 1 | 1.34 |
| *Aeromonas veronii CECT 4199T DSM* | 2 | 1.20 |
| *Agromyces cerinus ssp nitratus HKI 11532_DSM 8596T HKJ* | 1 | 1.24 |
| *Aromatoleum toluolicum T MPB* | 1 | 1.40 |
| *Candida glabrata 31 PSB* | 1 | 1.18 |
| *Clostridium tetani 1089_ATCC 10779 BOG* | 1 | 1.39 |
| *Escherichia coli ATCC 25922 CHB* | 1 | 1.17 |
| *Escherichia coli DH5alpha BRL* | 1 | 1.30 |
| *Hydrogenophaga flava B339 UFL* | 1 | 1.40 |
| *Lactobacillus crispatus DSM 20356 DSM* | 3 | 1.33 |
| *Lactobacillus crispatus DSM 20584T DSM* | 2 | 1.23 |
| *Lactobacillus kefiri DSM 20485 DSM* | 1 | 1.26 |
| *Lactobacillus mucosae DSM 13345T DSM* | 3 | 1.31 |
| *Neisseria meningitidis C1 2 PGM* | 2 | 1.29 |
| *Paraburkholderia fungorum LMG 20227T HAM* | 3 | 1.32 |
| *Staphylococcus lugdunensis DSM 4805 DSM* | 1 | 1.23 |
| ***Schistosoma_mansoni_sme_2_female_2 (2 worms)*** | **32** | **1.36** |
| *Aromatoleum toluolicum T MPB* | 1 | 1.29 |
| *Arthrobacter koreensis DSM 16760T DSM* | 1 | 1.29 |
| *Candida guilliermondii CBS 566 CBS* | 1 | 1.40 |
| *Candida tropicalis ATCC 13803 THL* | 3 | 1.32 |
| *Hydrogenophaga flava B339 UFL* | 2 | 1.47 |
| *Kandleria vitulina DSM 20405T DSM* | 12 | 1.36 |
| *Pichia occidentalis CBS 1910 CBS* | 1 | 1.42 |
| *Pseudomonas oryzihabitans DSM 6835T HAM* | 1 | 1.33 |
| *Serratia marcescens 13103_1 CHB* | 7 | 1.36 |
| *Staphylococcus lugdunensis DSM 4805 DSM* | 2 | 1.43 |
| *Staphylococcus warneri Mb18796_1 CHB* | 1 | 1.23 |
| ***Schistosoma_mansoni_sme_2_male_2 (2 worms)*** | **24** | **1.36** |
| *Arthrobacter parietis DSM 16404T DSM* | 2 | 1.30 |
| *Candida guilliermondii CBS 566 CBS* | 1 | 1.31 |
| *Candida lambica CBS 603 CBS* | 4 | 1.39 |
| *Corynebacterium urealyticum DSM 7109T DSM* | 2 | 1.42 |
| *Lactobacillus oligofermentans DSM 15709 DSM* | 1 | 1.38 |
| *Neisseria meningitidis C1 2 PGM* | 1 | 1.35 |
| *Pseudomonas aeruginosa DSM 50071T HAM* | 1 | 1.31 |
| *Sphingomonas paucimobilis B341 UFL* | 4 | 1.36 |
| *Staphylococcus aureus ssp aureus DSM 20232 DSM* | 2 | 1.37 |
| *Staphylococcus aureus ssp aureus DSM 346 DSM* | 3 | 1.35 |
| *Staphylococcus aureus ssp aureus DSM 4910 DSM* | 1 | 1.40 |
| *Streptomyces lavendulae B264 UFL* | 2 | 1.38 |
| ***Schistosoma_mansoni_sme_female_1 (1 worm)*** | **12** | **1.38** |
| *Candida tropicalis ATCC 13803 THL* | 2 | 1.31 |
| *Corynebacterium striatum 23086514 MLD* | 1 | 1.36 |
| *Escherichia coli DH5alpha BRL* | 3 | 1.43 |
| *Escherichia coli RV412_A1_2010_06a LBK* | 1 | 1.50 |
| *Hydrogenophaga flava B339 UFL* | 1 | 1.49 |
| *Lactobacillus crispatus DSM 20356 DSM* | 3 | 1.33 |
| *Pseudomonas putida DSM 50198 HAM* | 1 | 1.36 |
| ***Schistosoma_mansoni_sme_female_4 (2 worms)*** | **24** | **1.36** |
| *Halomonas elongata B582 UFL* | 1 | 1.36 |
| *Hydrogenophaga flava B339 UFL* | 3 | 1.35 |
| *Kandleria vitulina DSM 20405T DSM* | 9 | 1.39 |
| *Lactobacillus satsumensis DSM 16230T DSM* | 1 | 1.34 |
| *Pichia occidentalis CBS 1910 CBS* | 1 | 1.33 |
| *Pseudomonas oryzihabitans DSM 6835T HAM* | 1 | 1.33 |
| *Serratia marcescens 13103_1 CHB* | 5 | 1.35 |
| *Staphylococcus aureus ssp aureus DSM 20232 DSM* | 1 | 1.34 |
| *Staphylococcus lugdunensis DSM 4805 DSM* | 2 | 1.34 |
| ***Schistosoma_mansoni_sme_female_5 (2 worms)*** | **21** | **1.36** |
| *Agromyces mediolanus DSM 20152T DSM* | 1 | 1.28 |
| *Candida tropicalis ATCC 13803 THL* | 3 | 1.33 |
| *Halomonas elongata B582 UFL* | 1 | 1.35 |
| *Hydrogenophaga flava B339 UFL* | 8 | 1.43 |
| *Kandleria vitulina DSM 20405T DSM* | 4 | 1.33 |
| *Lactobacillus kitasatonis DSM 16761T DSM* | 1 | 1.25 |
| *Pseudomonas rhizosphaerae LMG 21640T HAM* | 1 | 1.36 |
| *Staphylococcus piscifermentans DSM 7373T DSM* | 1 | 1.27 |
| *Trichosporon ovoides CBS 7556T CBS* | 1 | 1.24 |
| ***Schistosoma_mansoni_sme_female_6 (2 worms)*** | **25** | **1.35** |
| *Aeromonas schubertii CECT 4240T DSM* | 1 | 1.26 |
| *Arthrobacter gandavensis DSM 15046T DSM* | 1 | 1.24 |
| *Candida tropicalis ATCC 13803 THL* | 4 | 1.33 |
| *Escherichia coli ATCC 25922 THL* | 1 | 1.31 |
| *Hydrogenophaga flava B339 UFL* | 4 | 1.41 |
| *Kandleria vitulina DSM 20405T DSM* | 2 | 1.42 |
| *Lactobacillus crispatus DSM 20356 DSM* | 1 | 1.26 |
| *Lactobacillus satsumensis DSM 16230T DSM* | 1 | 1.36 |
| *Pichia occidentalis CBS 1910 CBS* | 3 | 1.33 |
| *Serratia marcescens 13103_1 CHB* | 2 | 1.34 |
| *Staphylococcus piscifermentans DSM 7373T DSM* | 2 | 1.36 |
| *Streptomyces lavendulae B264 UFL* | 3 | 1.41 |
| ***Schistosoma_mansoni_sme_male_1 (1 worm)*** | **16** | **1.35** |
| *Campylobacter jejuni MB_6111_05 THL* | 1 | 1.33 |
| *Candida guilliermondii CBS 566 CBS* | 1 | 1.44 |
| *Corynebacterium jeikeium VA_3882_07_13 UKE* | 3 | 1.34 |
| *Hydrogenophaga flava B339 UFL* | 1 | 1.30 |
| *Neisseria meningitidis C1 2 PGM* | 1 | 1.32 |
| *Pseudomonas extremorientalis DSM 15824T HAM* | 3 | 1.34 |
| *Pseudomonas nitroreducens LMG 20221T HAM* | 1 | 1.30 |
| *Pseudomonas oryzihabitans DSM 6835T HAM* | 1 | 1.30 |
| *Sphingomonas paucimobilis B341 UFL* | 1 | 1.42 |
| *Staphylococcus aureus ssp aureus DSM 20232 DSM* | 3 | 1.40 |
| ***Schistosoma_mansoni_sme_male_4 (2 worms)*** | **1** | **1.50** |
| *Candida lambica CBS 603 CBS* | 1 | 1.50 |
| ***Schistosoma_mansoni_sme_male_4 (2 worms)*** | **15** | **1.38** |
| *Candida guilliermondii CBS 566 CBS* | 3 | 1.39 |
| *Candida lambica CBS 603 CBS* | 1 | 1.53 |
| *Clostridium baratii 1084_ATCC 25782 BOG* | 1 | 1.34 |
| *Cryptococcus neoformans ATCC 14116 THL* | 2 | 1.38 |
| *Enterococcus faecium VRE_PX_16086218 MLD* | 1 | 1.32 |
| *Lactobacillus fermentum DSM 20391 DSM* | 1 | 1.41 |
| *Sphingomonas paucimobilis B341 UFL* | 2 | 1.36 |
| *Sphingomonas sp B556 UFL* | 1 | 1.40 |
| *Staphylococcus aureus ssp aureus DSM 346 DSM* | 2 | 1.38 |
| *Staphylococcus auricularis DSM 20609 DSM* | 1 | 1.31 |
| ***Schistosoma_mansoni_sme_male_5 (2 worms)*** | **30** | **1.41** |
| *Agromyces italicus HKI 325_DSM 16388T HKJ* | 1 | 1.39 |
| *Candida dubliniensis CM 1 CBS* | 1 | 1.28 |
| *Candida lambica CBS 603 CBS* | 1 | 1.42 |
| *Cryptococcus neoformans ATCC 14116 THL* | 2 | 1.39 |
| *Escherichia coli DH5alpha BRL* | 1 | 1.63 |
| *Escherichia coli MB11464_1 CHB* | 2 | 1.56 |
| *Escherichia coli RV412_A1_2010_06a LBK* | 3 | 1.46 |
| *Helcococcus kunzii 13134278_2 MVD* | 1 | 1.34 |
| *Hydrogenophaga flava B339 UFL* | 3 | 1.31 |
| *no peaks found* | 6 | #DIV/0! |
| *Pseudomonas extremorientalis DSM 15824T HAM* | 1 | 1.32 |
| *Sphingomonas paucimobilis B341 UFL* | 2 | 1.37 |
| *Staphylococcus aureus ssp aureus DSM 346 DSM* | 1 | 1.33 |
| *Staphylococcus aureus ssp aureus DSM 4910 DSM* | 1 | 1.45 |
| *Staphylococcus carnosus ssp carnosus DSM 20501T DSM* | 1 | 1.29 |
| *Staphylococcus condimenti DSM 11675 DSM* | 1 | 1.48 |
| *Staphylococcus epidermidis 6b_s ESL* | 1 | 1.40 |
| *Staphylococcus epidermidis ATCC 12228 THL* | 1 | 1.51 |
| ***Schistosoma_mansoni_sme_male_6 (2 worms)*** | **16** | **1.38** |
| *Candida guilliermondii CBS 566 CBS* | 3 | 1.36 |
| *Cryptococcus neoformans ATCC 14116 THL* | 1 | 1.47 |
| *Escherichia coli DH5alpha BRL* | 1 | 1.43 |
| *Hydrogenophaga flava B339 UFL* | 1 | 1.33 |
| *Lactobacillus oligofermentans DSM 15709 DSM* | 1 | 1.38 |
| *Paracoccus versutus B352 UFL* | 3 | 1.39 |
| *Sphingomonas paucimobilis B341 UFL* | 1 | 1.44 |
| *Staphylococcus aureus ssp aureus DSM 20232 DSM* | 1 | 1.33 |
| *Staphylococcus aureus ssp aureus DSM 4910 DSM* | 1 | 1.33 |
| *Staphylococcus condimenti DSM 11675 DSM* | 2 | 1.39 |
| *Staphylococcus epidermidis 10547 CHB* | 1 | 1.34 |
| ***Schistosoma_mansoni_smR_female_1 (2 worms)*** | **24** | **1.25** |
| *Achromobacter xylosoxidans MU_15202_1 CHB* | 3 | 1.25 |
| *Aeromonas veronii CECT 4199T DSM* | 4 | 1.26 |
| *Bacteroides fragilis MB_9009_05 THL* | 2 | 1.33 |
| *Brevundimonas aurantiaca DSM 4731T HAM* | 2 | 1.26 |
| *Clostridium novyi A 1025_NCTC 538 BOG* | 1 | 1.21 |
| *Enterococcus faecalis 105652 LDW* | 1 | 1.17 |
| *Lactobacillus delbrueckii ssp lactis DSM 20355 DSM* | 1 | 1.20 |
| *Lactobacillus ultunensis DSM 16048 DSM* | 2 | 1.27 |
| *Methylobacterium rhodesianum MB99 UFL* | 1 | 1.27 |
| *Neisseria animaloris CCUG 53090 CCUG* | 1 | 1.26 |
| *Staphylococcus carnosus ssp carnosus DSM 20501T DSM* | 1 | 1.18 |
| *Staphylococcus carnosus ssp utilis DSM 11677 DSM* | 1 | 1.21 |
| *Staphylococcus pseudintermedius CCUG 50530 CCUG* | 1 | 1.21 |
| *Trichophyton benhamiae DSM 6916 DSM* | 2 | 1.31 |
| *Xanthomonas campestris DSM 50852 DSM* | 1 | 1.24 |
| ***Schistosoma_mansoni_smR_female_2 (2 worms)*** | **32** | **1.25** |
| *Clostridium chauvoei 1076_ATCC 10092T BOG* | 1 | 1.29 |
| *Flavobacterium saccharophilum DSM 1811T HAM* | 3 | 1.25 |
| *Lactobacillus equi DSM 15833T DSM* | 1 | 1.25 |
| *Lactobacillus gastricus DSM 16046 DSM* | 1 | 1.19 |
| *Lactobacillus malefermentans DSM 5705T DSM* | 1 | 1.20 |
| *Lactobacillus paralimentarius DSM 13961 DSM* | 5 | 1.26 |
| *Lactobacillus plantarum DSM 13273 DSM* | 1 | 1.23 |
| *Monascus ruber 2016_3_D IFS* | 1 | 1.37 |
| *Nocardia sp MB_9090_05 THL* | 1 | 1.14 |
| *Pseudomonas aeruginosa 19955_1 CHB* | 2 | 1.21 |
| *Pseudomonas citronellolis DSM 50332T HAM* | 1 | 1.26 |
| *Staphylococcus epidermidis 6b_s ESL* | 1 | 1.31 |
| *Streptomyces badius B192 UFL* | 13 | 1.25 |
| ***Schistosoma_mansoni_smR_female_3 (2 worms)*** | **32** | **1.24** |
| *Achromobacter xylosoxidans MU_15202_1 CHB* | 2 | 1.27 |
| *Acidovorax konjaci DSM 7481T HAM* | 1 | 1.24 |
| *Bacteroides fragilis MB_9009_05 THL* | 4 | 1.23 |
| *Candida guilliermondii CBS 566 CBS* | 3 | 1.20 |
| *Cryptococcus neoformans 29 PSB* | 1 | 1.26 |
| *Lactobacillus amylolyticus DSM 11664T DSM* | 9 | 1.28 |
| *Lactobacillus sakei ssp carnosus DSM 15740 DSM* | 1 | 1.19 |
| *Paraburkholderia phymatum LMG 21445T HAM* | 2 | 1.18 |
| *Pseudomonas aeruginosa 19955_1 CHB* | 1 | 1.21 |
| *Staphylococcus equorum ssp equorum DSM 20675 DSM* | 1 | 1.12 |
| *Staphylococcus pseudintermedius 472 RLT* | 5 | 1.26 |
| *Streptomyces griseus B261 UFL* | 2 | 1.27 |
| ***Schistosoma_mansoni_SmR_female_4*** | **24** | **1.23** |
| *Aromatoleum toluvorans Td21 MPB* | 1 | 1.24 |
| *Bacillus funiculus DSM 15141T DSM* | 2 | 1.33 |
| *Bacteroides fragilis MB_9009_05 THL* | 2 | 1.22 |
| *Brevundimonas aurantiaca DSM 4731T HAM* | 1 | 1.43 |
| *Flavobacterium piscis DICM09_00412 VSV* | 1 | 1.16 |
| *Flavobacterium saccharophilum DSM 1811T HAM* | 2 | 1.28 |
| *Helicobacter canis CIP 104753T CBB* | 2 | 1.18 |
| *Kandleria vitulina DSM 20405T DSM* | 1 | 1.21 |
| *Lactobacillus amylolyticus DSM 11664T DSM* | 2 | 1.14 |
| *Lactobacillus sharpeae DSM 20504 DSM* | 1 | 1.20 |
| *Nocardia sp MB_9090_05 THL* | 1 | 1.26 |
| *Ralstonia pickettii 21323_1 CHB* | 1 | 1.19 |
| *Sphingomonas sp B399 UFL* | 1 | 1.19 |
| *Staphylococcus equorum ssp equorum DSM 20675 DSM* | 2 | 1.23 |
| *Staphylococcus pseudintermedius 472 RLT* | 2 | 1.24 |
| *Streptomyces griseus B261 UFL* | 2 | 1.27 |
| ***Schistosoma_mansoni_SmR_female_5*** | **28** | **1.28** |
| *Achromobacter xylosoxidans MU_15202_1 CHB* | 1 | 1.25 |
| *Alistipes onderdonkii DSM 19147T DSM* | 1 | 1.23 |
| *Brevundimonas aurantiaca DSM 4731T HAM* | 2 | 1.31 |
| *Flavobacterium saccharophilum DSM 1811T HAM* | 8 | 1.29 |
| *Lactobacillus amylolyticus DSM 11664T DSM* | 1 | 1.26 |
| *Lactobacillus plantarum DSM 13273 DSM* | 1 | 1.26 |
| *Methylobacterium rhodesianum MB99 UFL* | 1 | 1.27 |
| *Sphingomonas paucimobilis B341 UFL* | 1 | 1.35 |
| *Staphylococcus aureus ATCC 33591 THL* | 1 | 1.30 |
| *Staphylococcus pseudintermedius 472 RLT* | 1 | 1.32 |
| *Staphylococcus saprophyticus ssp saprophyticus DSM 4853 DSM* | 1 | 1.20 |
| *Streptomyces griseus B261 UFL* | 8 | 1.29 |
| *Trichophyton benhamiae DSM 6916 DSM* | 1 | 1.15 |
| ***Schistosoma_mansoni_smR_male_1 (2 worms)*** | **28** | **1.27** |
| *Acinetobacter junii DSM 6964T HAM* | 4 | 1.24 |
| *Bacillus flexus 100331_30 USP* | 1 | 1.32 |
| *Exiguobacterium sp[4] 10_0147905_008_01 LGL* | 1 | 1.25 |
| *Janthinobacterium lividum CIP 106720T HAM* | 1 | 1.28 |
| *Lactobacillus paralimentarius DSM 13238T DSM* | 2 | 1.30 |
| *Methylobacterium rhodesianum MB96 UFL* | 4 | 1.33 |
| *Methylobacterium rhodesianum MB99 UFL* | 6 | 1.28 |
| *Mycobacterium gordonae 0714 BSI* | 1 | 1.20 |
| *Nocardia sp N133 IBS* | 4 | 1.26 |
| *Prevotella melaninogenica 110706_G7 LUMC* | 1 | 1.29 |
| *Rhodococcus erythropolis DSM 12789 DSM* | 1 | 1.20 |
| *Sphingomonas sp B399 UFL* | 1 | 1.28 |
| *Sphingomonas wittichii DSM 6014T HAM* | 1 | 1.27 |
| ***Schistosoma_mansoni_smR_male_2 (2 worms)*** | **32** | **1.26** |
| *Aerococcus sanguinicola CCUG 61975 CCUG* | 1 | 1.21 |
| *Bacteroides fragilis 15470 PNU* | 1 | 1.20 |
| *Bacteroides fragilis MB_5088_05 THL* | 1 | 1.29 |
| *Candida krusei ATCC 6258 THL* | 1 | 1.23 |
| *Capnocytophaga granulosa DSM 11449T DSM* | 3 | 1.23 |
| *Cryptococcus neoformans ATCC 14116 THL* | 1 | 1.20 |
| *Geotrichum capitatum CBS 598_83 CBS* | 2 | 1.31 |
| *Janthinobacterium lividum CIP 106720T HAM* | 9 | 1.27 |
| *Lactobacillus antri DSM 16041T DSM* | 1 | 1.30 |
| *Lactobacillus antri DSM 16042 DSM* | 2 | 1.24 |
| *Lactobacillus paracasei ssp paracasei DSM 20020 DSM* | 1 | 1.24 |
| *Lactobacillus paracasei ssp paracasei DSM 20244 DSM* | 1 | 1.23 |
| *Lactobacillus paracasei ssp paracasei DSM 46331 DSM* | 3 | 1.27 |
| *Neisseria meningitidis C1 2 PGM* | 1 | 1.26 |
| *Nocardia sp N133 IBS* | 4 | 1.26 |
| ***Schistosoma_mansoni_smR_male_3 (1 worm)*** | **25** | **1.26** |
| *Bacillus alcalophilus B446 UFL* | 1 | 1.33 |
| *Bacteroides fragilis MB_5088_05 THL* | 2 | 1.23 |
| *Bacteroides fragilis MB_9009_05 THL* | 1 | 1.34 |
| *Cryptococcus neoformans ATCC 14116 THL* | 3 | 1.23 |
| *Hathewaya histolytica 1036_NCTC 503T BOG* | 1 | 1.32 |
| *Lactobacillus paracasei ssp paracasei DSM 46331 DSM* | 5 | 1.26 |
| *Lactobacillus perolens DSM 12745 DSM* | 1 | 1.31 |
| *Mycobacterium gordonae 0714 BSI* | 1 | 1.22 |
| *Nocardia sp N133 IBS* | 6 | 1.27 |
| *Paeniglutamicibacter sulfureus DSM 20167T DSM* | 1 | 1.17 |
| *Sphingobacterium mizutaii DSM 11724T HAM* | 1 | 1.22 |
| *Streptococcus equinus DSM 20558T DSM* | 1 | 1.25 |
| *Streptococcus gallolyticus ssp macedonicus DSM 15879T DSM* | 1 | 1.26 |
| ***Schistosoma_mansoni_SmR_male_4*** | **28** | **1.26** |
| *Arthrobacter stackebrandtii DSM 16005T DSM* | 1 | 1.27 |
| *Bacillus drentensis DSM 15600T DSM* | 3 | 1.29 |
| *Bacillus hemicellulosilyticus DSM 16731T DSM* | 1 | 1.21 |
| *Candida krusei ATCC 6258 THL* | 1 | 1.26 |
| *Capnocytophaga sp G18141 IBS* | 1 | 1.25 |
| *Cryptococcus neoformans ATCC 14116 THL* | 5 | 1.29 |
| *Enterobacter cloacae 13159_1 CHB* | 1 | 1.27 |
| *Gluconobacter oxydans ssp oxydans B544 UFL* | 1 | 1.19 |
| *Lactobacillus paracasei ssp paracasei DSM 46331 DSM* | 4 | 1.24 |
| *Lactobacillus paralimentarius DSM 13238T DSM* | 8 | 1.27 |
| *Methylobacterium rhodesianum MB99 UFL* | 1 | 1.19 |
| *Nocardia sp N133 IBS* | 1 | 1.28 |
| ***Schistosoma_mansoni_SmR_male_5*** | **24** | **1.27** |
| *Acinetobacter junii DSM 6964T HAM* | 1 | 1.36 |
| *Bacillus drentensis DSM 15600T DSM* | 1 | 1.27 |
| *Cryptococcus neoformans ATCC 14116 THL* | 1 | 1.23 |
| *Gluconobacter oxydans ssp oxydans B544 UFL* | 1 | 1.21 |
| *Lactobacillus fuchuensis DSM 14342 DSM* | 2 | 1.34 |
| *Lactobacillus paracasei ssp paracasei DSM 20006 DSM* | 1 | 1.26 |
| *Lactobacillus paracasei ssp paracasei DSM 20020 DSM* | 1 | 1.21 |
| *Lactobacillus paracasei ssp paracasei DSM 46331 DSM* | 6 | 1.27 |
| *Lactobacillus paralimentarius DSM 13238T DSM* | 4 | 1.29 |
| *Methylobacterium rhodesianum MB99 UFL* | 1 | 1.37 |
| *Neisseria meningitidis C1 2 PGM* | 1 | 1.22 |
| *Nocardia sp N133 IBS* | 1 | 1.29 |
| *Phoma herbarum DSM 63184 DSM* | 1 | 1.26 |
| *Pseudomonas stutzeri B367 UFL* | 1 | 1.24 |
| *Staphylococcus fleurettii DSM 13212T DSM* | 1 | 1.20 |
| ***Schistosoma_mansoni_smR_male+female_10*** | **23** | **1.25** |
| *Achromobacter xylosoxidans MU_15202_1 CHB* | 1 | 1.30 |
| *Bacillus funiculus DSM 15141T DSM* | 3 | 1.26 |
| *Bacteroides fragilis MB_9009_05 THL* | 2 | 1.34 |
| *Blastomonas ursincola DSM 9006T HAM* | 1 | 1.22 |
| *Capnocytophaga granulosa DSM 11449T DSM* | 1 | 1.25 |
| *Cellulomonas massiliensis DSM 25695T DSM* | 1 | 1.28 |
| *Clostridium ramosum CCUG 45030 CCUG* | 1 | 1.25 |
| *Lactobacillus antri DSM 16042 DSM* | 1 | 1.28 |
| *Lactobacillus paracasei ssp paracasei DSM 46331 DSM* | 1 | 1.28 |
| *Lactobacillus paracasei ssp paracasei DSM 8741 DSM* | 1 | 1.26 |
| *Lactobacillus perolens DSM 12745 DSM* | 1 | 1.22 |
| *Methylobacterium extorquens MB125 UFL* | 1 | 1.18 |
| *Methylobacterium fujisawaense B235 UFL* | 1 | 1.25 |
| *Methylobacterium rhodesianum MB99 UFL* | 1 | 1.20 |
| *Mycobacterium gordonae 0714 BSI* | 1 | 1.22 |
| *Neisseria meningitidis C1 2 PGM* | 1 | 1.21 |
| *Rothia amarae DSM 15839T DSM* | 1 | 1.22 |
| *Staphylococcus pseudintermedius 472 RLT* | 1 | 1.24 |
| *Staphylococcus pseudintermedius CCUG 50530 CCUG* | 1 | 1.19 |
| *Streptomyces griseus B261 UFL* | 1 | 1.26 |
| ***Schistosoma_mansoni_smR_male+female_11*** | **25** | **1.26** |
| *Achromobacter xylosoxidans MU_15202_1 CHB* | 1 | 1.22 |
| *Aeromonas veronii CECT 4199T DSM* | 1 | 1.20 |
| *Bacillus funiculus DSM 15141T DSM* | 2 | 1.26 |
| *Bacteroides fragilis MB_9009_05 THL* | 3 | 1.26 |
| *Blastomonas ursincola DSM 9006T HAM* | 1 | 1.24 |
| *Cryptococcus humicola CBS 571T CBS* | 1 | 1.31 |
| *Helicobacter canis CIP 104753T CBB* | 1 | 1.25 |
| *Lactobacillus amylophilus DSM 20533T DSM* | 1 | 1.33 |
| *Lactobacillus fuchuensis DSM 14342 DSM* | 2 | 1.26 |
| *Lactobacillus parabuchneri DSM 5708 DSM* | 1 | 1.20 |
| *Lactobacillus paralimentarius DSM 13238T DSM* | 1 | 1.26 |
| *Methylobacterium rhodesianum MB96 UFL* | 2 | 1.25 |
| *Paeniglutamicibacter sulfureus DSM 20167T DSM* | 1 | 1.33 |
| *Pseudomonas putida B401 UFL* | 1 | 1.23 |
| *Sphingomonas aurantiaca DSM 14748T HAM* | 3 | 1.22 |
| *Staphylococcus carnosus ssp utilis DSM 11677 DSM* | 1 | 1.34 |
| *Staphylococcus pseudintermedius CCUG 50530 CCUG* | 1 | 1.32 |
| *Streptococcus equinus DSM 20558T DSM* | 1 | 1.23 |
| ***Schistosoma_mansoni_smR_male+female_6*** | **24** | **1.26** |
| *Acinetobacter junii DSM 6964T HAM* | 2 | 1.31 |
| *Alistipes onderdonkii DSM 19147T DSM* | 1 | 1.23 |
| *Bacillus funiculus DSM 15141T DSM* | 3 | 1.23 |
| *Bacillus jeotgali DSM 18226T DSM* | 1 | 1.23 |
| *Cellulomonas massiliensis DSM 25695T DSM* | 1 | 1.19 |
| *Cryptococcus saitoi CBS 1734 CBS* | 1 | 1.21 |
| *Gluconobacter oxydans ssp oxydans B544 UFL* | 1 | 1.26 |
| *Lactobacillus amylolyticus DSM 11664T DSM* | 1 | 1.30 |
| *Lactobacillus perolens DSM 12745 DSM* | 2 | 1.23 |
| *Methylobacterium rhodesianum MB93 UFL* | 1 | 1.28 |
| *Neisseria meningitidis C1 2 PGM* | 2 | 1.24 |
| *Nocardia sp N133 IBS* | 2 | 1.26 |
| *Paeniglutamicibacter sulfureus DSM 20167T DSM* | 2 | 1.37 |
| *Phoma herbarum DSM 63184 DSM* | 2 | 1.28 |
| *Pseudomonas aeruginosa 19955_1 CHB* | 2 | 1.26 |
| ***Schistosoma_mansoni_smR_male+female_7*** | **32** | **1.25** |
| *Achromobacter xylosoxidans MU_15202_1 CHB* | 2 | 1.26 |
| *Acinetobacter junii DSM 6964T HAM* | 2 | 1.28 |
| *Acinetobacter parvus DSM 16617T HAM* | 3 | 1.20 |
| *Aerococcus sanguinicola CCUG 64841 CCUG* | 1 | 1.21 |
| *Bacillus funiculus DSM 15141T DSM* | 6 | 1.23 |
| *Cryptococcus neoformans 29 PSB* | 1 | 1.29 |
| *Lactobacillus amylovorus DSM 20531T DSM* | 1 | 1.25 |
| *Lactobacillus fuchuensis DSM 14342 DSM* | 1 | 1.27 |
| *Lactobacillus mucosae DSM 13345T DSM* | 1 | 1.23 |
| *Lactobacillus paracasei ssp paracasei DSM 46331 DSM* | 1 | 1.26 |
| *Lactobacillus paralimentarius DSM 13238T DSM* | 2 | 1.26 |
| *Lactobacillus perolens DSM 12745 DSM* | 2 | 1.18 |
| *Neisseria meningitidis C1 2 PGM* | 4 | 1.28 |
| *Nocardia sp N133 IBS* | 1 | 1.41 |
| *Phoma herbarum DSM 63184 DSM* | 1 | 1.20 |
| *Staphylococcus pseudintermedius 472 RLT* | 1 | 1.26 |
| *Staphylococcus pseudintermedius CCUG 50530 CCUG* | 1 | 1.21 |
| *Streptomyces griseus B261 UFL* | 1 | 1.25 |
| ***Schistosoma_mansoni_smR_male+female_8*** | **22** | **1.26** |
| *Achromobacter xylosoxidans MU_15202_1 CHB* | 3 | 1.33 |
| *Acinetobacter junii DSM 6964T HAM* | 2 | 1.23 |
| *Acinetobacter parvus DSM 16617T HAM* | 1 | 1.23 |
| *Aerococcus sanguinicola CCUG 64841 CCUG* | 1 | 1.19 |
| *Bacillus alcalophilus B446 UFL* | 1 | 1.31 |
| *Cellulomonas massiliensis DSM 25695T DSM* | 1 | 1.23 |
| *Lactobacillus fuchuensis DSM 14342 DSM* | 2 | 1.31 |
| *Lactobacillus paracasei ssp paracasei DSM 46331 DSM* | 2 | 1.26 |
| *Lactobacillus perolens DSM 12745 DSM* | 2 | 1.26 |
| *Mycobacterium gordonae 0714 BSI* | 1 | 1.33 |
| *Neisseria meningitidis C1 2 PGM* | 1 | 1.26 |
| *Nocardia sp N133 IBS* | 1 | 1.24 |
| *Phoma herbarum DSM 63184 DSM* | 1 | 1.25 |
| *Staphylococcus pseudintermedius CCUG 50530 CCUG* | 1 | 1.17 |
| *Streptomyces griseus B261 UFL* | 2 | 1.21 |
| ***Schistosoma_mansoni_smR_male+female_9*** | **22** | **1.22** |
| *Acinetobacter parvus DSM 16617T HAM* | 1 | 1.26 |
| *Bacillus alcalophilus B446 UFL* | 1 | 1.24 |
| *Bacillus funiculus DSM 15141T DSM* | 2 | 1.22 |
| *Candida catenulata DSM 70136 DSM* | 1 | 1.21 |
| *Lactobacillus amylolyticus DSM 11664T DSM* | 1 | 1.29 |
| *Lactobacillus antri DSM 16042 DSM* | 1 | 1.25 |
| *Lactobacillus fuchuensis DSM 14342 DSM* | 1 | 1.21 |
| *Lactobacillus paralimentarius DSM 13238T DSM* | 1 | 1.17 |
| *Lactobacillus zeae DSM 20178T DSM* | 2 | 1.22 |
| *Methylobacterium rhodesianum MB96 UFL* | 1 | 1.20 |
| *Neisseria meningitidis C1 2 PGM* | 3 | 1.22 |
| *Saccharopolyspora erythraea HKI 184 HKJ* | 1 | 1.10 |
| *Staphylococcus pseudintermedius 472 RLT* | 2 | 1.25 |
| *Staphylococcus pseudintermedius CCUG 50530 CCUG* | 1 | 1.37 |
| *Streptomyces albus B262 UFL* | 1 | 1.25 |
| *Streptomyces griseus B261 UFL* | 1 | 1.23 |
| *Tsukamurella inchonensis DSM 43246 DSM_2* | 1 | 1.08 |
| ***Schistosoma_mansoni_SmR_male+femele_1*** | **27** | **1.27** |
| *Acinetobacter junii DSM 6964T HAM* | 2 | 1.26 |
| *Bacillus funiculus DSM 15141T DSM* | 1 | 1.29 |
| *Bacillus jeotgali DSM 18226T DSM* | 1 | 1.28 |
| *Bacteroides fragilis MB_5088_05 THL* | 2 | 1.27 |
| *Bacteroides fragilis MB_9009_05 THL* | 3 | 1.27 |
| *Geotrichum capitatum CBS 598_83 CBS* | 1 | 1.32 |
| *Janthinobacterium lividum CIP 106720T HAM* | 2 | 1.33 |
| *Lactobacillus delbrueckii ssp bulgaricus DSM 20081T DSM* | 1 | 1.19 |
| *Lactobacillus fuchuensis DSM 14341 DSM* | 1 | 1.23 |
| *Lactobacillus fuchuensis DSM 14342 DSM* | 2 | 1.29 |
| *Lactobacillus paracasei ssp paracasei DSM 20020 DSM* | 3 | 1.29 |
| *Lactobacillus paracasei ssp paracasei DSM 2649 DSM* | 1 | 1.25 |
| *Neisseria animaloris CCUG 53090 CCUG* | 3 | 1.26 |
| *Staphylococcus epidermidis 6b_s ESL* | 1 | 1.18 |
| *Staphylococcus pseudintermedius 472 RLT* | 2 | 1.30 |
| *Thauera mechernichensis Tl1 MPB* | 1 | 1.19 |
| ***Schistosoma_mansoni_SmR_male+femele_2*** | **29** | **1.28** |
| *Achromobacter xylosoxidans MU_15202_1 CHB* | 1 | 1.24 |
| *Acinetobacter junii DSM 6964T HAM* | 2 | 1.33 |
| *Acinetobacter parvus DSM 16617T HAM* | 1 | 1.28 |
| *Bacillus jeotgali DSM 18226T DSM* | 1 | 1.33 |
| *Bacteroides fragilis MB_9009_05 THL* | 3 | 1.22 |
| *Lactobacillus delbrueckii ssp delbrueckii DSM 20074T DSM* | 1 | 1.30 |
| *Lactobacillus paracasei ssp paracasei DSM 20244 DSM* | 1 | 1.24 |
| *Lactobacillus paracasei ssp paracasei DSM 46331 DSM* | 1 | 1.19 |
| *Neisseria meningitidis C1 2 PGM* | 5 | 1.27 |
| *Pseudomonas oryzihabitans DSM 6835T HAM* | 1 | 1.30 |
| *Staphylococcus pseudintermedius 472 RLT* | 7 | 1.31 |
| *Staphylococcus pseudintermedius CCUG 50530 CCUG* | 5 | 1.27 |
| ***Schistosoma_mansoni_SmR_male+femele_3*** | **29** | **1.31** |
| *Acinetobacter junii DSM 6964T HAM* | 1 | 1.38 |
| *Alistipes indistinctus DSM 22520T DSM* | 1 | 1.24 |
| *Bacillus funiculus DSM 15141T DSM* | 3 | 1.27 |
| *Bacillus jeotgali DSM 18226T DSM* | 1 | 1.32 |
| *Bacteroides fragilis MB_9009_05 THL* | 1 | 1.34 |
| *Cryptococcus saitoi CBS 1734 CBS* | 1 | 1.30 |
| *Lactobacillus delbrueckii ssp bulgaricus DSM 20081T DSM* | 1 | 1.28 |
| *Lactobacillus fuchuensis DSM 14342 DSM* | 1 | 1.35 |
| *Neisseria meningitidis C1 2 PGM* | 1 | 1.30 |
| *Nocardia cyriacigeorgica 120619_21 HUA* | 1 | 1.30 |
| *Nocardia sp N133 IBS* | 1 | 1.25 |
| *Pseudomonas putida B411 UFL* | 1 | 1.22 |
| *Ralstonia sp B484 UFL* | 1 | 1.31 |
| *Staphylococcus pseudintermedius 472 RLT* | 6 | 1.36 |
| *Staphylococcus pseudintermedius CCUG 50530 CCUG* | 8 | 1.29 |
| ***Schistosoma_mansoni_SmR_male+femele_4*** | **30** | **1.31** |
| *Achromobacter xylosoxidans MU_15202_1 CHB* | 1 | 1.34 |
| *Acinetobacter junii DSM 6964T HAM* | 1 | 1.36 |
| *Bacillus funiculus DSM 15141T DSM* | 6 | 1.36 |
| *Bacillus jeotgali DSM 18226T DSM* | 4 | 1.31 |
| *Bacteroides fragilis MB_9009_05 THL* | 1 | 1.23 |
| *Chryseobacterium ureilyticum CF_09 HUA* | 1 | 1.27 |
| *Enterococcus avium LMG 12171 LMG* | 1 | 1.26 |
| *Lactobacillus kalixensis DSM 16044 DSM* | 1 | 1.28 |
| *Lactobacillus paracasei ssp paracasei DSM 46331 DSM* | 1 | 1.32 |
| *Neisseria meningitidis C1 2 PGM* | 2 | 1.25 |
| *Neisseria subflava CCUG 29402 CCUG* | 1 | 1.21 |
| *Pseudomonas putida B401 UFL* | 2 | 1.32 |
| *Sphingomonas parapaucimobilis DSM 7463T DSM* | 1 | 1.21 |
| *Staphylococcus pseudintermedius 472 RLT* | 7 | 1.33 |
| ***Schistosoma_mansoni_SmR_male+femele_5*** | **26** | **1.28** |
| *Acinetobacter junii DSM 6964T HAM* | 2 | 1.34 |
| *Acinetobacter parvus DSM 16617T HAM* | 1 | 1.30 |
| *Bacillus funiculus DSM 15141T DSM* | 2 | 1.24 |
| *Bacillus jeotgali DSM 18226T DSM* | 2 | 1.34 |
| *Bacteroides fragilis MB_9009_05 THL* | 1 | 1.42 |
| *Cryptococcus neoformans ATCC 14116 THL* | 1 | 1.30 |
| *Lactobacillus delbrueckii ssp bulgaricus DSM 20081T DSM* | 5 | 1.28 |
| *Lactobacillus kalixensis DSM 16044 DSM* | 1 | 1.28 |
| *Lactobacillus paracasei ssp paracasei DSM 20020 DSM* | 2 | 1.35 |
| *Lactobacillus paracasei ssp paracasei DSM 20244 DSM* | 1 | 1.29 |
| *Lactobacillus ultunensis DSM 16048 DSM* | 2 | 1.26 |
| *Leuconostoc pseudomesenteroides DSM 5624 DSM* | 1 | 1.21 |
| *Neisseria meningitidis C1 2 PGM* | 1 | 1.22 |
| *Nocardia cyriacigeorgica 120619_21 HUA* | 1 | 1.20 |
| *Nocardia sp N133 IBS* | 1 | 1.24 |
| *Pseudomonas aeruginosa 19955_1 CHB* | 1 | 1.34 |
| *Streptococcus dysgalactiae ssp dysgalactiae DSM 20662T DSM* | 1 | 1.14 |
| **Total** | **1655** | **1.31** |
