# Supplementary figures and images for: Matrix-assisted laser desorption/ionization time-of-flight mass spectrometry for differential identification of adult Schistosoma worms
Source: Parasit Vectors. 2023 Jan 19;16:20. doi: 10.1186/s13071-022-05604-0 (PMC9854196; doi:10.1186/s13071-022-05604-0)

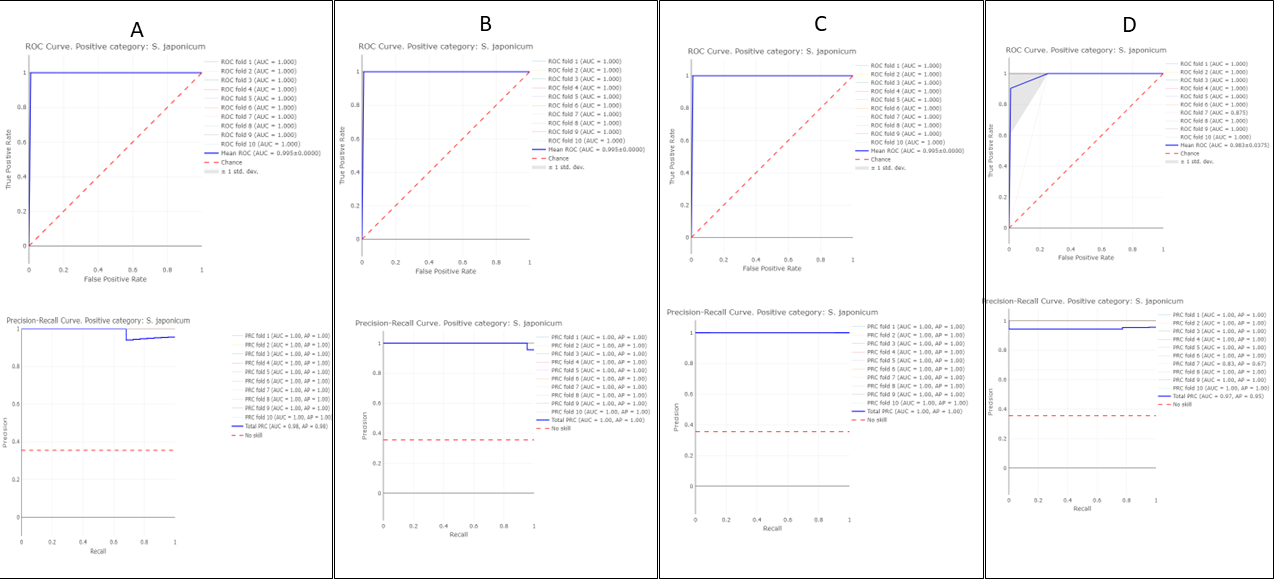

Supplement: Supplementary file 2 — Additional file 2: Figure S1. Receiver operating characteristic (ROC) and precision-recall (PR) curves and their related areas under the curves [area under receiver operating characteristic curve (AUROC) and area under the precision-recall curve (AUPR)] for Schistosoma species classification using supervised machine learning (ML) algorithms. a Support vector machine, b partial least square–discriminant analysis (PLS-DA), c Random Forest (RF), and d k-nearest neighbor (KNN). [file 13071_2022_5604_MOESM2_ESM.tif]
